# Nanoscale, Voltage-Driven Application of Bioactive Substances onto Cells with Organized Topography

**DOI:** 10.1016/j.bpj.2015.11.017

**Published:** 2016-01-05

**Authors:** Sophie Schobesberger, Peter Jönsson, Andrey Buzuk, Yuri Korchev, Jennifer Siggers, Julia Gorelik

**Affiliations:** 1 Department of Medicine, Imperial College London, London, United Kingdom; 2 Department of Bioengineering, Imperial College London, London, United Kingdom; 3 Department of Chemistry, Lund University, Lund, Sweden

## Abstract

With scanning ion conductance microscopy (SICM), a noncontact scanning probe technique, it is possible both to obtain information about the surface topography of live cells and to apply molecules onto specific nanoscale structures. The technique is therefore widely used to apply chemical compounds and to study the properties of molecules on the surfaces of various cell types. The heart muscle cells, i.e., the cardiomyocytes, possess a highly elaborate, unique surface topography including transverse-tubule (T-tubule) openings leading into a cell internal system that exclusively harbors many proteins necessary for the cell’s physiological function. Here, we applied isoproterenol into these surface openings by changing the applied voltage over the SICM nanopipette. To determine the grade of precision of our application we used finite-element simulations to investigate how the concentration profile varies over the cell surface. We first obtained topography scans of the cardiomyocytes using SICM and then determined the electrophoretic mobility of isoproterenol in a high ion solution to be −7 × 10^−9^ m^2^/V s. The simulations showed that the delivery to the T-tubule opening is highly confined to the underlying Z-groove, and especially to the first T-tubule opening, where the concentration is ∼6.5 times higher compared to on a flat surface under the same delivery settings. Delivery to the crest, instead of the T-tubule opening, resulted in a much lower concentration, emphasizing the importance of topography in agonist delivery. In conclusion, SICM, unlike other techniques, can reliably deliver precise quantities of compounds to the T-tubules of cardiomyocytes

## Introduction

In cell physiology, increasing attention is paid to the specific location of receptors and proteins on the highly sensitive surface membrane of biological samples like neurons (1), bacilli (2), platelets (3), and heart muscle cells (4, 5). The specific location of molecules on the cell surface may underlie differences in their activity in health, and their positioning can change under disease conditions (5). Such differences and changes cannot be studied efficiently with, for example, whole-cell stimulation of receptors (5) or activation of channels (6), but requires a more thorough method of investigation. We have previously investigated the intricately structured beating cells of the heart, the cardiomyocytes. These cells exhibit unique structural features of nanoscale dimension with immense importance to their physiological function. The external topography of cardiomyocytes can be categorized into crests and surface grooves (Z-grooves) in which the transverse-tubule (T-tubule) openings reside and protrude deep into the cell body (7). Due to their small size and their spatial arrangement, these T-tubules generate a highly regulated and very unique environment for the ions needed in excitation-contraction coupling, the process of translating an electrical impulse into the physiological beating response of cardiomyocytes (8). In healthy cardiomyocytes, the T-tubule openings exclusively house a plethora of receptors and ion channels including the *β*2 adrenergic receptors (*β*_2_ARs), the L-type Ca^2+^ channels, and the Na^+^/Ca^2+^ exchanger, which are vital for cardiomyocyte function (5). The *β*_2_ARs can be activated by adrenaline or its chemical analog, isoproterenol (9). Scanning ion conductance microscopy (SICM) (10) is a noncontact scanning technique (11), which has often been used to investigate the nanoscale structure of the surface of live cells (3, 4, 5). This is achieved by measuring the ion current between an electrode inside the nanopipette and an electrode in the surrounding bath solution (11). The SICM nanopipette can furthermore be used to apply chemical agonists onto different surface structures of the sample with high precision (5). Our group have previously used air pressure to displace the electrolyte solution in the nanopipette for such application (5). However, during the relatively slow air-pressure applications, the nanopipette can be blocked. Hence, our group sought a faster and more reliable method of application. This could be achieved by switching the direction of the electrical current applied over the nanopipette and using this to deliver the agonist (12, 13). However, the amplitude (amount of molecules being delivered) and precision of this application method have not been investigated. This knowledge is vital for interpreting the results obtained from delivery experiments correctly, as well as for future research avenues that SICM could open up. No other technique to our knowledge holds as much promise for the investigation and manipulation of small and precise structures such as, for instance, a single T-tubule. Hence, the aim of this work is to use finite-element simulations to quantify the delivery of isoproterenol to heart cell structures.

## Materials and Methods

Cardiomyocytes were plated on a dish and perfused with a physiological (pH 7.3) electrolyte solution (144 mM NaCl, 5 mM KCl, and 1 mM MgCl_2_; see the Supporting Material for details on the preparation of the cells). After acquiring the surface topography with SICM, the nanopipette, prefilled with 50 *μ*M Isoproterenol, was directed over a T-tubule opening. Then the electrical holding potential of −200 mV was changed to +400 mV to expel the chemical agonist by electroosmotic forces. The electroosmotic drift velocity is independent of the charge of the molecules in solution and arises from mobile cations in the electric double layer next to the pipette wall. For the negatively charged glass wall we use a value for the electroosmotic mobility, *μ*_eo_, of 1.4 × 10^−8^ m^2^/V s, which approximately corresponds to the value in an electrolyte solution with 150 mM Na^+^ (13). This will in turn give rise to a convective liquid flow in the direction of the electric field. However, since isoproterenol is negatively charged, the delivery will be counteracted by an electrophoretic drift component (14). The electrophoretic mobility, *μ*_ep_, of isoproterenol was measured in the aforementioned electrolyte solution at 25°C using phase analysis light scattering (PALS), with a NanoBrook ZetaPALS (Brookhaven Instruments, Stockport, United Kingdom). From the Smoluchowski drift-diffusion equation, this resulted in an average value of −7 × 10^−9^ ± 0.2 × 10^−9^ m^2^/V s (mean value ± 1 SD).

The molecular flow rate out of the pipette, corresponding to the number of molecules of Isoproteronol leaving the pipette per second, can be shown to be approximately given by (13)

(1) $$Q_{\text{tot}}=c_{0}\left(\mu_{\text{ep}}+\mu_{\text{eo}}\right)\pi R_{0}\;\tan\left(\theta \right)\Delta \Psi ,$$

where *c*_0_ is the concentration of molecules in the bulk of the pipette, *R*_0_ is the inner tip radius of the pipette, *θ* is the inner half-cone angle, and ΔΨ is the voltage drop over the pipette. When the molecules leave the pipette they will be rapidly diluted due to diffusion. It can be shown that for a flat surface, the concentration at distances of *R* = (*x*^2^ + *y*^2^ + (*z* − *h*)^2^)^0.5^ >> *R*_0_ from the tip of the pipette at (0,0,*z* = *h*) approximately varies according to the expression given by

(2) $$c\left(x,y\right)=c_{0}\left(2-\exp\left(-Q_{\text{tot}}/\left(4\pi c_{0}D\sqrt{x^{2}+y^{2}+{\left(z-h\right)}^{2}}\right)\right)-\exp\left(-Q_{\text{tot}}/\left(4\pi c_{0}D\sqrt{x^{2}+y^{2}+{\left(z+h\right)}^{2}}\right)\right)\right),$$

where *D* is the diffusivity of the molecules and *h is* the distance between the tip of the pipette and the surface. When *c* << *c*_0_, Eq. 2 simplifies to the expression on the flat surface at *z* = 0:

(3) $$c\left(x,y\right)\approx c_{0}\frac{\left(\mu_{\text{ep}}+\mu_{\text{eo}}\right)R_{0}\;\tan\left(\theta \right)\Delta \Psi }{D\sqrt{x^{2}+y^{2}+h^{2}}}.$$

**Figure 1:**
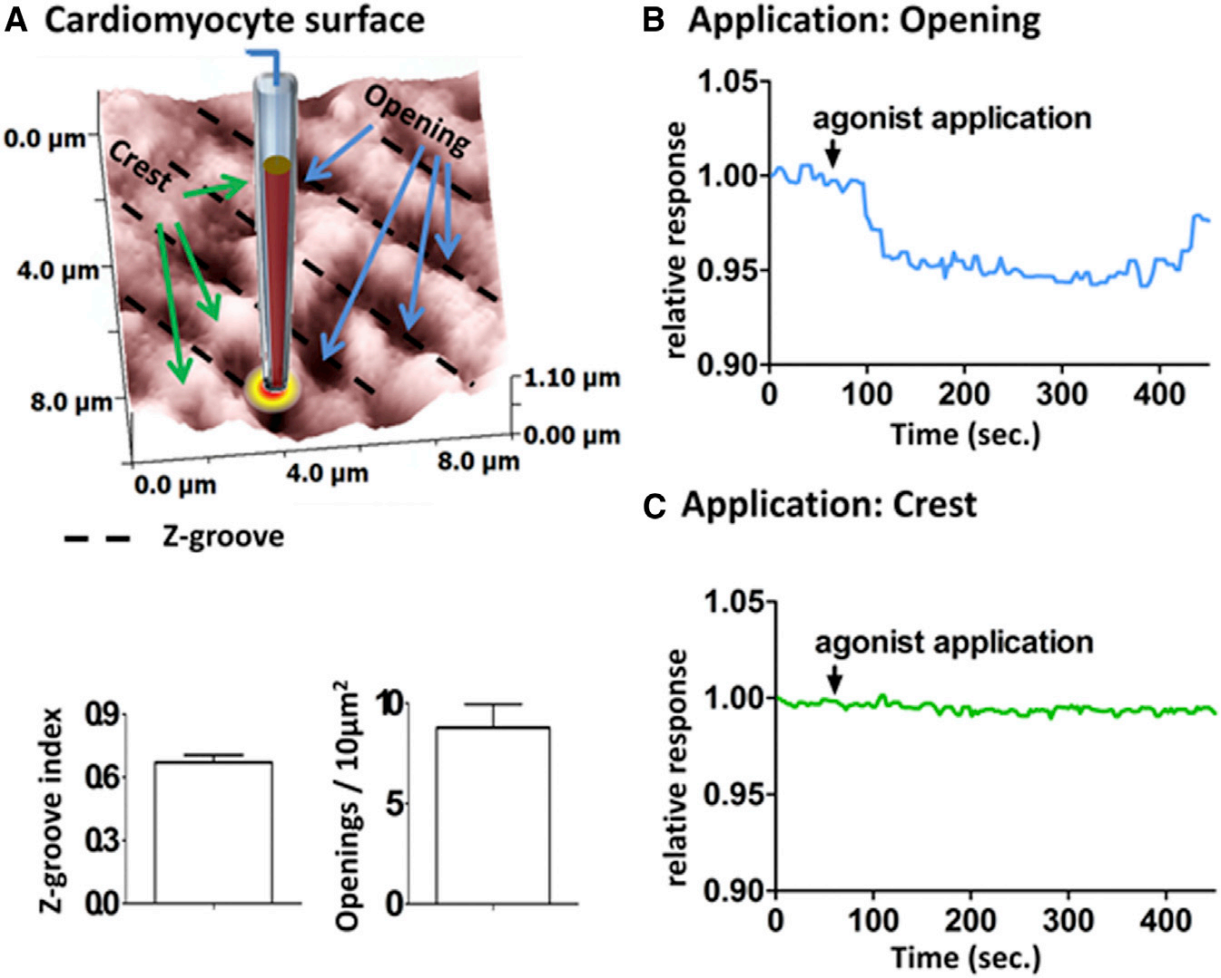
(*A*) (*Upper*) A 10 × 10 *μ*m^2^ SICM surface scan, schematically showing isoproterenol application into an opening on the surface of a cardiomyocyte. Z-grooves are indicated by black dotted lines, openings by blue arrows, and crests by green arrows. (*Lower*) Summary of the respective Z-groove index, an indicator of how many Z-grooves are present, which is defined by determining the length of all Z-grooves and dividing this value by the maximally extrapolated length possible on the 10 × 10 *μ*m^2^ scan (7) (*lower left*), and the number of openings on the 10 × 10 *μ*m^2^ area (*lower right*) (*N* = 10). (*B* and *C*) Graphs showing the cell internal *β*_2_AR-dependent second messenger response to agonist application into the T-tubule opening (*B*) and onto the crest of the cell surface (*C*), obtained by moving the pipette to the respective coordinates on the computer-generated SICM scan. To see this figure in color, go online.

**Figure 2:**
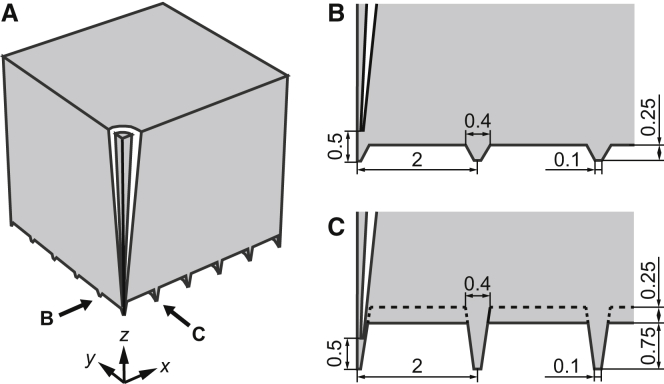
The geometry used in the finite-element simulations of a cardiomyocyte. (*A*) The simulation geometry consists of a cube with a side length of 10 *μ*m, in which parts have been subtracted to make up the Z-grooves and T-tubule openings as well as the pipette. (*B*) A zoom-in of the *yz*-plane at *x* = 0 together with dimensions of the simulation geometry. (*C*) A zoom-in of the *xz*-plane at *y* = 0 together with dimensions of the simulation geometry. The pipette has an inner tip radius of 50 nm, an outer tip radius of 100 nm, and an inner half-cone angle of 3°. All distances are given in micrometers.

The concentration thus scales approximately inversely with the distance to the point (0,0) and increases linearly with the radius of the pipette tip and the applied voltage. However, it is not known what the magnitude of the concentration outside the pipette will be, and how the concentration profile will look, when the surface is not flat. To investigate this, we first measured the topography of typical cardiomyocyte surface structures using SICM. From the obtained surface scans (Fig. 1), the number of T-tubule openings and Z-grooves on the cardiomyocytes was determined and averaged to obtain the necessary parameters for the simulation geometry (Fig. 2). Finite-element simulations using the program COMSOL Multiphysics 5.0 (COMSOL, Burlington, MA) were performed to estimate the delivered amount of Isoproterenol, using the parameter values in Table 1. Additional information about the details of the numerical simulations and on the mathematical boundary conditions can be found in the Supporting Material.

## Results

Under experimental conditions the *β*_2_AR-dependent second messenger signal in the cardiomyocytes only changed when isoproterenol was applied into the T-tubule opening on the cell surface and not on the crest area between the Z-grooves, as can be seen in Fig. 1, *B* and *C* (a detailed description of the SICM and the *β*_2_AR-dependent second messenger signal measurements are given in the Supporting Material). To estimate the amount of isoproterenol delivered, we used finite-element simulations to establish the approximate amount of the molecule isoproterenol being delivered from a nanopipette to underlying cardiomyocyte structures and determined the delivered concentration profile over the highly structured heart cell surfaces. To define the parameters of the model, we determined a representative model of the cell surface, the pipette dimensions, and the ligand’s electrophoretic mobility.

The diameter of the T-tubule opening at the primary application site was measured to be ∼400 nm. Based on the aforementioned values, a representative model of healthy cardiomyocytes was constructed from the SICM images (Figs. 1 *A* and 2). The simulation geometry consists of a 10 *μ*m^3^ cube with parts subtracted to construct

1) grooves 0.4 *μ*m wide and 1 *μ*m high along the *y*-direction, spaced 2 *μ*m apart;
2) grooves 0.4 *μ*m wide and 0.25 *μ*m high in the *x*-direction, spaced 2 *μ*m apart; and
3) a pipette with an inner tip radius of 50 nm, an outer radius of 100 nm, and an inner half-cone angle of 3°.

**Figure 3:**
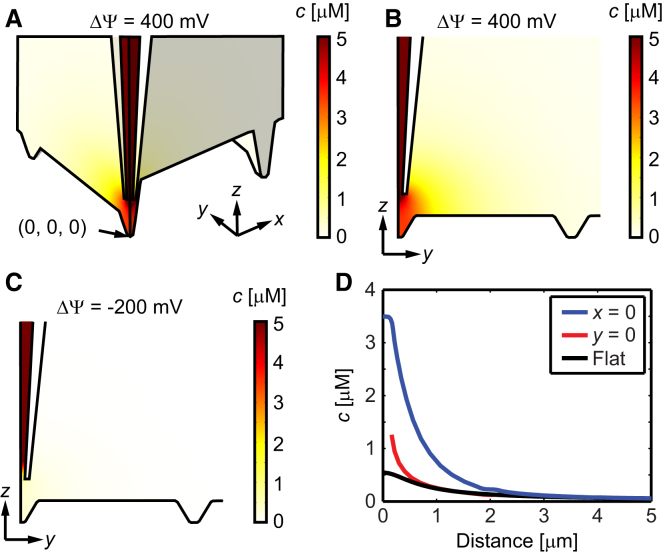
Delivery of isoproterenol to a T-tubule opening. (*A*) 3D image showing the simulated concentration of isoproterenol when delivering with ΔΨ = 400 mV. The concentration in the bulk of the pipette is 50 *μ*M. (*B* and *C*) 2D zoom-in at the plane *x* = 0 showing the concentration distribution at ΔΨ = 400 mV and −200 mV, respectively. (*D*) Line profiles of the concentration at ΔΨ = 400 mV along the lower edge at *x* = 0 (*blue*) and *y* = 0 (*red*) as a function of the distances *y* and *x*, respectively, from the point (*x*, *y*, *z*) = (0, 0, 0). The black line is the corresponding value for a flat surface at *z* = 0 using Eqs. 1 and 2. To see this figure in color, go online.

The simulated concentration outside the pipette when delivering with a voltage of 400 mV is shown in Fig. 3 together with the situation when delivering with ΔΨ = −200 mV. For the positive delivery voltage 7 × 10^5^ molecules/s of isoproterenol are leaving the pipette, which is in good agreement with the predicted value from Eq. 1. Thus, approximately the same amount of isoproterenol is leaving the pipette when delivering into the Z-groove as when delivering to a flat surface, which is the case as long as the flow resistance outside the pipette is smaller than the flow resistance in the interior of the pipette (12). However, the concentration profiles outside the pipette will be different for the two cases, as discussed below. There is also a flow of molecules out of the pipette due to diffusion when applying ΔΨ = −200 mV. This value is ∼5 × 10^4^ molecules/s, which is an order of magnitude lower than the value at ΔΨ = 400 mV, but is not zero. It can therefore be advisable to withdraw the pipette from the sample between delivery time points to avoid excessive delivery, and thus stimulus, of the cardiomyocytes. Note also, that the concentration in the bulk of the pipette is 50 *μ*M, but that the color scale in Fig. 3 is between 0 and 5 *μ*M to better visualize the concentration profile outside the pipette. Details about the finite-element simulations are given in the Supporting Material.

The molecular flow of isoproterenol is initially guided along the groove at *x* = 0 in the *y*-direction. The concentration along the lower edge at *x* = 0 and the lower edge at *y* = 0 is shown in Fig. 3 *D*, where the concentration is also compared to the corresponding value for a flat surface, *h* = 0.5 *μ*m below the pipette, using the theoretical expressions in Eqs. 1 and 2. The distance is given as the value of *y* for the edge at *x* = 0 and as the value of *x* for the edge at *y* = 0.

The concentration at the T-tubule opening at (*x*, *y*, *z*) = (0, 0, 0) is ∼3.5 *μ*M, which is ∼6.5 times higher than the concentration for a flat surface at the same distance to the pipette. The reason for this behavior is that the molecules are initially limited to diffusion along the length of the Z-groove, which results in a slower decrease of the concentration in this direction. At larger distances, the molecules start to diffuse in all directions again and the concentration approaches that of a flat surface. In fact, the concentration at the T-tubule opening in the second groove at (*x*, *y*, *z*) = (2 *μ*m, 0, 0) is only ∼10% different from the value for a flat surface. This value is ∼30 times lower than the concentration at the T-tubule opening at (*x*, *y*, *z*) = (0, 0, 0). The concentration for the second T-tubule opening in the groove at (*x*, *y*, *z*) = (0, 2 *μ*m, 0) is higher due to the guiding effect of the groove, but it is still ∼15 times lower compared to the concentration over the first opening (at *x* = *y* = 0), indicating that the delivery is mainly limited to the T-tubule opening beneath the pipette. Another possibility is that the Z-groove focuses the flow out of the pipette, and that this effect is causing the increase in concentration at the underlying T-tubule opening. However, we have previously observed that the concentration profile outside nanopipettes, at a distance larger than one pipette radius from the tip, is generally dominated by diffusion and that the convective term here, as a first approximation, can be neglected (13). This was found also to be true for delivery to a Z-groove, since setting the convective term equal to zero outside the pipette only resulted in a 20% drop in the concentration at the T-tubule opening, significantly less than the 6.5 times decrease when delivering to a flat surface.

**Figure 4:**
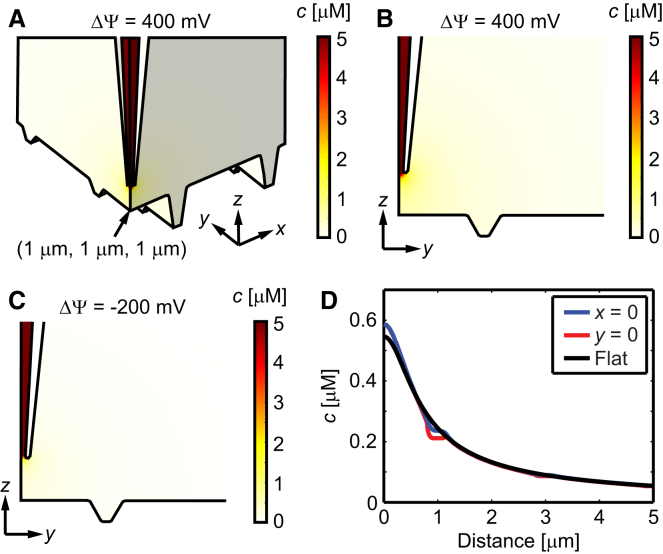
Delivery of isoproterenol to the crest between T-tubule openings. (*A*) 3D image showing the concentration of isoproterenol when delivering with ΔΨ = 400 mV. The concentration in the bulk of the pipette is 50 *μ*M. (*B* and *C*) 2D zoom-in at the plane *x* = 1 *μ*m showing the concentration distribution at ΔΨ = 400 mV and ΔΨ = −200 mV, respectively. (*D*) Line profiles of the concentration along the lower edge at *x* = 1 *μ*m (*blue*) and *y* = 1 *μ*m (*red*) as a function of the distances *y* and *x*, respectively, from the point (*x*, *y*, *z*) = (1, 1, 1) *μ*m. The black line is the corresponding value for a flat surface using Eqs. 1 and 2 (relative to the point (1, 1, 1) *μ*m). To see this figure in color, go online.

When instead delivering to the crest between the openings (*x* = *y* = 1 *μ*m in Fig. 2), *h* = 500 nm above the surface, the concentration in the nearest T-tubule is ∼20 times lower compared to when delivering directly to the T-tubule (Fig. 4). This is well in line with the observation that there is a response only if delivering agonist to the T-tubule opening and not to the crest, as shown in Fig. 1, *B* and *C*.

The concentration profile will change for different values of the parameters in Table 1, but will approximately scale with ΔΨ, *R*_0_ (assuming *R*_1_ = 2*R*_0_), *θ*, *D*, and *μ*_ep_ according to the expression in Eq. 3 when *c* << *c*_0_ (see Fig. S3). For example, for a pipette with a 20% larger radius the concentration will be 20% larger if all other parameters are kept constant and, similarly, the concentration will scale roughly linearly with the applied voltage under the condition that *c* << *c*_0_. Simulations were also made where the distance between the tip of the pipette and the T-tubule opening, *h*, was varied, but otherwise under the same conditions as summarized in Table 1. The results from these simulations are shown in Fig. S4, indicating that the difference in concentration at the T-tubule opening, compared to delivery onto a flat surface, is largest when the pipette is close to the T-tubule opening. When the pipette retracts further from the Z-groove, the concentration profile approaches that for delivery to a flat surface. A COMSOL Multiphysics 5.0 file of the simulations described in this work is included in the Supporting Materials.

The charge distribution from an electrical double layer close to the walls of the pipette has so far been neglected in the simulations of the electric field. However, for small nanopipettes this can give rise to a significant change in both the electric field and the electroosmotic flow through the pipette (17). Additional simulations were therefore made to investigate this effect on the current system, where the concentration of Na^+^ and Cl^-^ in the nanopipette was simulated and used as the charge distribution for the electric field simulations in the nanopipette. COMSOL Multiphysics was again used for this following the procedure outlined by Ivanov et al. (17). The dimensions of the pipette are given in Table 1 and the size of the simulation geometry was scaled up by a factor of 4 to take into account the larger pipette size compared to the nanopipettes used by Ivanov et al. Only the situation with a pipette far from the underlying surface was investigated, which was considered adequate to obtain an estimate of how the electric field at the tip of the pipette and the total osmotic flow are affected by the electrical double layer. For the pipette walls, a surface charge of *σ* = −18 mC/m^2^ was assumed (corresponding to a *ζ* potential of *ζ* = −20 mV (17)), which gives an electroosmotic mobility of 1.4 × 10^−8^ m^2^/V s. The concentration of Na^+^ and Cl^−^ was set to 150 mM, and the diffusivities of Na^+^ and Cl^−^ used were *D*_Na_^+^ = 1.33 × 10^−9^ m^2^/s and *D*_Cl_- = 2.03 × 10^−9^ m^2^/s, respectively (18). Under these conditions, only modest differences in the electroosmotic flow out of the pipette were found. The total electroosmotic flow rate out of the pipette deviated by <2% when including the effect of the electrical double layer, and the deviation in the electric field in the *z*-direction, along the axis of the pipette, was <10%. Thus, for the current situation, where the inner tip radius of the nanopipette is ∼60 times larger than the Debye length at the salt concentration used, the effect of the electrical double layer on the electric field is only minor. However, this effect might be more pronounced for smaller pipettes, or when using low salt concentrations.

Our results show that delivery to a single opening on the cell surface can be made repeatedly and reliably, but also that the effect of the topography on the delivery can be significant. This is of special importance in the unique case of cardiomyocytes, as their structures hold the key to their proper physiological function.

## Discussion

An increasing number of investigations highlight the importance of compartment-specific signaling of this cell type, and investigation and manipulation of single compartments will become crucial for fully understanding their physiological and pathophysiological regulation. So far, to our knowledge, SICM is the only method that enables us to perform noninvasive experiments that are as localized as a single T-tubule opening. The analytical verification of the application method presented here encourages a range of further local application experiments. The technique allows for delivery of essentially any compound to multiple structured cell types with a precision determined by the pipette dimensions and the potential compound charge. In the case of cardiology, this concept could be applied to delivering fluorescent dyes and investigating diffusion dynamics within the T-tubule network, delivering universal and specific agonists as well as ions, for example, Ca^2+^ to monitor induced Ca^2+^ release. The concept could also be expanded to local application after membrane sealing by the nanopipette, which would avoid diffusion of the agonist into the extracellular space and lead to higher concentrations at the structure. Therefore, we present a universal approach to quantify local application and to adjust such parameters as precision, speed, and ultimately concentration through modulating the current and pipette structure, which promises to yield important data in cardiology and beyond.

## Conclusion

Even though the simulations presented here were for the specific case of isoproterenol delivery to cardiomyocytes, we propose that they can also be applied and made useful for other cell types and agonist delivery applications, as they allow the quantification, and possible optimization, of application processes that deal with nanoscale structures that evade experimental determination.

## Author Contributions

Research design was carried out by S.S., P.J., J.S., and J.G.; research was performed by S.S. and P.J.; graphics were generated by S.S., P.J., A.B., and Y.K.; analytic tools were contributed by P.J., Y.K., and J.G.; data were analyzed by S.S., P.J., and A.B.; and the article was written by S.S., P.J., A.B., J.S., and J.G.

## Figures and Tables

**Table 1:** Parameters and Values for Modeling the Delivery of Isoproterenol

Name	Description	Value
*R*_0_	inner tip radius	50 nm
*R*_1_	outer tip radius	100 nm
*θ*	inner half-cone angle	3°
*h*	pipette-surface distance	500 nm
*D*	diffusivity of ISO (15)	6.7 × 10^−10^ m^2^/s
*μ*_ep_	electrophoretic mobility of ISO	−7 × 10^−9^ m^2^/V s
*μ*_eo_	electroosmotic mobility in the pipette (13)	1.4 × 10^−8^ m^2^/V s
*c*_0_	concentration of ISO inside the pipette	50 *μ*M
ΔΨ	applied voltage over the pipette	400 mV

ISO, isoproterenol.

## Supporting References

References (19, 20) appear in the Supporting Material.

## Supporting Material

Document S1. Supporting Materials and Methods, Figures S1–S4, and Table S1

Document S2. Article plus Supporting Material
