# Supplementary material for: Nanoscale, Voltage-Driven Application of Bioactive Substances onto Cells with Organized Topography
Source: Biophys J. 2016 Jan 5;110(1):141–6. doi: 10.1016/j.bpj.2015.11.017 (PMC4805872; doi:10.1016/j.bpj.2015.11.017)
Supplement: Document S1. Supporting Materials and Methods, Figures S1–S4, and Table S1 [file mmc1.pdf]

Biophysical Journal, Volume *110*

Supplemental Information

Nanoscale, Voltage-Driven Application of Bioactive Substances onto Cells with Organized Topography

Sophie Schobesberger, Peter Jönsson, Andrey Buzuk, Yuri Korchev, Jennifer Siggers, and Julia Gorelik

Precise, nanoscale, voltage-driven application of bio-active substances onto cardiomyocyte surface with organized topography

Sophie Schobesberger‡¤, Peter Jönsson‡†, Andrey Buzuk¤, Yuri Korchev¤, Jennifer Siggers¶, Julia Gorelik¤*

¤Department of Medicine, Imperial College London, London, United Kingdom

¶Department of Bioengineering, Imperial College London, London, United Kingdom

† Department of Chemistry, Lund University, Lund, Sweden

Author Contributions

‡These authors contributed equally.

Correspondence to: Julia Gorelik, PhD (e-mail: j.gorelik@imperial.ac.uk, Tel: +44 (0)20 7594 2736, Fax: 44 (0)20 7594 3653), Department of Cardiac Medicine, National Heart and Lung Institute, Imperial College, ICTEM 4th floor, London W12 0NN, UK

Supporting Material:

Contents

1. Research Animals 1
2. Cardiomyocyte cell isolation and culture 1
3. Cardiomyocyte transfection with a FRET biosensor to detect the cyclic nucleotide cAMP 2
4. Determination of cell surface structures using Scanning Ion Conductance Microscopy 2
5. Measurements of the β adrenergic receptor response using FRET 2
6. Measurements of the electrophoretic mobility of Isoproterenol 3

1. Research Animals

All procedures were carried out in compliance with the standards for the care and use of animal subjects as stated in the Guide of the Care and Use of Laboratory Animals (NIH publication No. 85–23, revised 1996) and the requirements of the UK Home Office (ASPA1986 Amendments Regulations 2012) incorporating the EU directive *2010/63/EU*. Protocols were approved by the Animal Care and Use Committee of Imperial College London.

2. Cardiomyocyte cell isolation and culture

Cardiomyocytes were obtained from the left ventricle of excised, adult Sprague-Dawley rat hearts via Langendorff perfusion and enzymatic digestion as described previously (1). For cell culture glass bottom dishes (MatTeK corporation, Ashland, USA) were coated with laminin and isolated cardiomyocytes were plated on the dishes and incubated at 5% CO_2 in modified M199 (Invitrogen, UK) culture medium containing per 500 mL bovine serum

albumin (0.5 g/L), creatine (5 mM/L), taurine (5 mM/L), L-ascorbic acid (100 mM/L), carnitine (2 mM), and penicillin/streptomycin (100 mM/L).

3. Cardiomyocyte transfection with a FRET biosensor to detect the cyclic nucleotide cAMP

To detect the cyclic nucleotide cAMP a Förster Resonance Energy Transfer (FRET) biosensor was used. The cAMP sensor Epac2-camps is made of the cAMP-binding domain of the Epac protein together with the donor fluorophore Cyan Fluorescent Protein (CFP) and the acceptor fluorophore Yellow Fluorescent Protein (YFP) attached at either end of the binding domain. Upon binding of cAMP the sensor undergoes a conformational change which brings the acceptor fluorophore apart from the donor fluorophore and the FRET energy exchange drops, which is recorded as a decrease in the fluorescent signal ratio of YFP to CFP. Cells were infected with the adeno-associated virus containing the Epac2-camps sensor and cultured for 48h as described previously (2).

4. Determination of cell surface structures using Scanning Ion Conductance Microscopy

For the visualization of the surface membrane topography of cardiomyocytes the Scanning Ion Conductance Microscope (SICM) in the "hopping" mode was used with nanopipettes of ~100 MΩ resistance as sensitive probes, as described previously (3). In short the SICM allows for a three-dimensional nanoscale topographic layout of the surface structure of live samples, such as T-tubules and crests on cardiomyocytes, to be obtained. This is achieved by measuring the ion current between a reference electrode inside the electrolyte-filled nanopipette, which serves as a nanoprobe, and a ground electrode, which is positioned in the sample dish; the feedback control mechanism keeps the distance between the pipette and the sample constant while the nanoprobe is scanning the sample. The displacement of the pipette is recorded and forms a three-dimensional image. The physiological electrolyte solution (pH 7.3) contained NaCl (144 mM), KCl (5 mM), HEPES (10 mM) and $MgCl_2$ (1 mM).

5. Measurements of the β adrenergic receptor response using FRET

All experiments were performed at room temperature (20-22°C) with the cardiomyocytes expressing the Epac2-camps FRET sensor being pre-incubated with CGP20712A (100 nM), a β_1AR receptor blocker. Topographical images (10×10 µm) were obtained by SICM to visualise the cell surface structure and distinguish between the T-tubule openings and the crest structures, in order to detect local β_2AR dependent cAMP FRET signals. β_2ARs were then stimulated locally via the SICM nanopipette being positioned either over a T-tubule opening or over a crest area. This was done by placing the pipette at defined coordinates according to the previously obtained surface scan, turning off the automatic feedback control that keeps the nanopipette at a constant distance (2500 nm when scanning cardiomyocytes) and lowering down the nanopipette until it is only 500 nm from the chosen surface structure. After establishing a baseline signal for 100 s, Isoproterenol (50 µM) was applied through the nanopipette by changing the electrical potential from -200 mV to 400 mV. The FRET sensor

was excited at 436 nm and the emission, detected with 535/40 nm and 480/30 nm filters, was recorded in two images simultaneously every 5 s with a Hamamatsu ORCA ER camera and the Micro-Manager 1.4 programme (4). The imaging system necessary for this was built around a Nikon TE2000 microscope and has been described previously (2). The schematic of the experiment is shown in **Supporting Figure 1**.

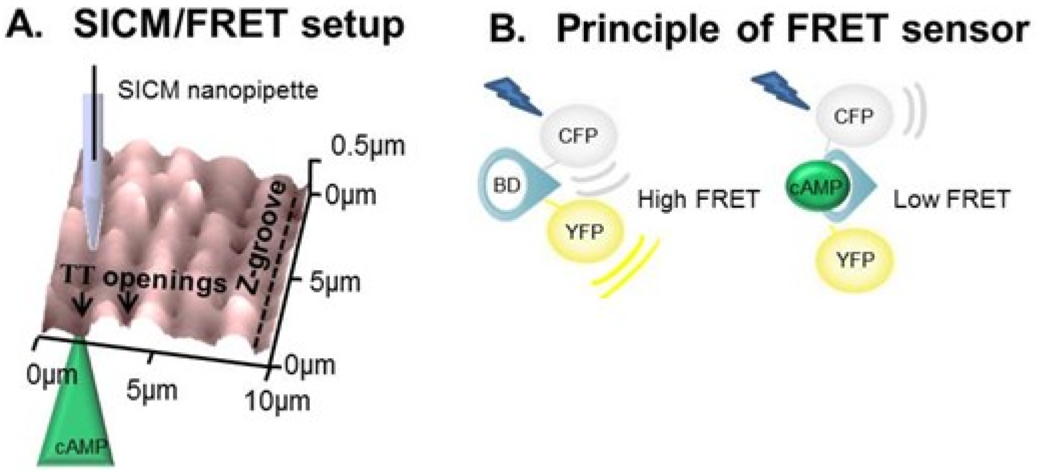

Supporting Figure 1. (A) Schematic of local β_2AR stimulation through a SICM pipette onto the surface of a healthy cardiomyocyte after using SICM to map the topography. **(B)** The principle of cAMP level measurements with the Epac2-camps FRET sensor; once cAMP binds the binding domain (BD) of the Epac protein a change in conformation of the FRET sensor will lead to a decrease in the FRET signal.

6. Measurements of the electrophoretic mobility of Isoproterenol

Measuring electrophoretic mobility can be achieved through Electrophoretic Light Scattering (ELS) techniques, which are based on the fact that moving particles which are hit by laser light will scatter the light according to their size and velocity due to, for example, Brownian motion or motion elicited by electrical fields. The scattered laser light can be detected and compared to a reference laser beam in order to determine the shift in the laser light frequency and with it the properties of the particles. Phase Analysis Light Scattering (PALS) is an advancement of the conventional ELS techniques and uses pulsatile laser beams and determines the motion and size of particles by the thereby generated phase shift of the detected light. By applying pulses only onto small fields PALS does not generate problematic alterations in temperature and does not destroy or change the properties of the sample. The NanoBrook machine used to carry out the PALS measurements furthermore facilitates sample testing in high salt buffer solutions with high conductance as was done here for Isoproterenol. Measurements of the electrophoretic mobility of Isoproterenol in physiological buffer (pH 7.3) containing NaCl (144 mM), KCl (5 mM), HEPES (10 mM) and $MgCl_2$ (1 mM) were conducted by Dr. Dan Clarke, on behalf of the company Brookhaven Instruments, UK. For the measurements the sample was analysed in 5 consecutive runs using a NanoBrook ZetaPALS machine (model: NanoBrook Omni, Brookhaven Instruments Corporation, UK) and phase analysis light scattering (PALS). The correlation-function of the phase shift from which the electrophoretic mobility was determined via the Smoluchowski drift-diffusion equation is shown below in Supporting Figure 2.

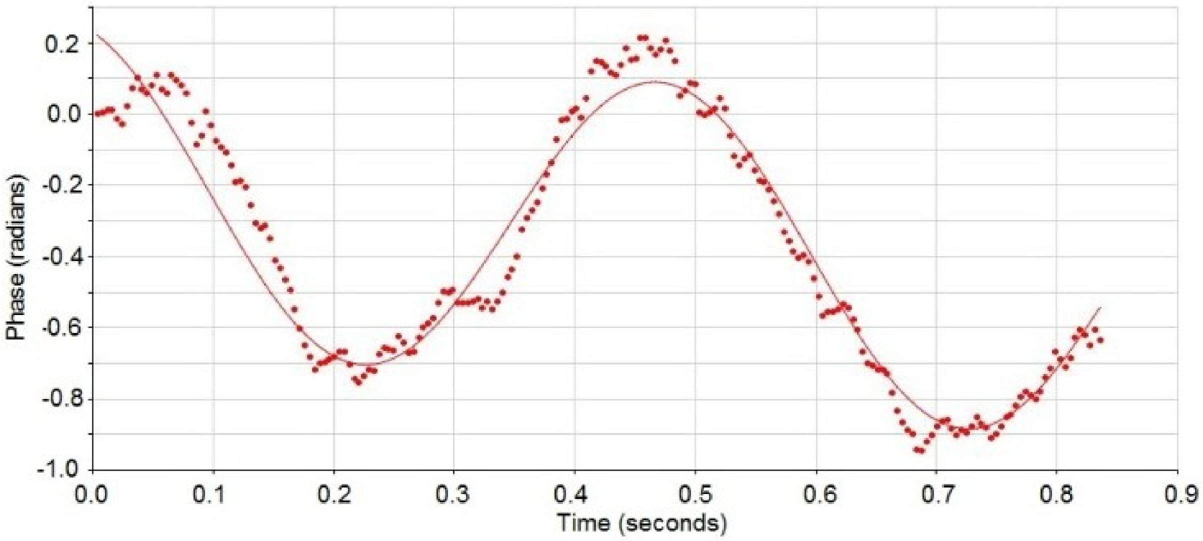

Supporting Figure 2. Correlation-function used to determine the electrophoretic mobility of Isoproterenol in physiological buffer using PALS.

7. Details regarding the numerical simulations

The program COMSOL Multiphysics® 5.0 (COMSOL, Inc.) was used to solve for the concentration of Isoproterenol delivered from the pipette due to a voltage drop, $\Delta\Psi$, applied over the pipette. The time it takes to reach steady state is much faster than the time scales normally used for the delivery and stationary equations can therefore be used. The following three sets of equations were solved:

(Electrostatics) $$\nabla^2\Psi = 0 \quad \text{(S1)}$$

(Creeping flow) $$-\nabla p + \eta\nabla^2\boldsymbol{u} = 0 \quad \text{(S2a)}$$

$$\nabla \cdot \boldsymbol{u} = 0 \quad \text{(S2b)}$$

(Transport of diluted species) $$\nabla \cdot \boldsymbol{J} = 0 \quad \text{(S3a)}$$

$$\boldsymbol{J} = -D\nabla c + c\left(\boldsymbol{u} - \mu_{\text{ep}}\nabla\Psi\right) \quad \text{(S3b)}$$

where Ψ is the electric potential, p the hydrostatic pressure, $\boldsymbol{u}$ the liquid flow vector, η (= 1 mPa s) the viscosity of the liquid and $\boldsymbol{J}$ the molecular flux of Isoproterenol. Equation S1 gives the electric field in the pipette, and is solved for first. Next, Eq. S2 is solved to determine the electroosmotic flow in the system, where the determined electric field $\boldsymbol{E} = -\nabla\Psi$ is used as input value. The concentration of Isoproterenol is finally determined by solving Eq. S3 with the already simulated values of $\boldsymbol{u}$ and Ψ as input values to determine the amount of Isoproterenol delivered. The boundary conditions used in the simulations are given in Supporting Table 1 with the parameter values in Table 1 in the main text. Note that the electrophoretic and electroosmotic mobility has opposite signs, and that the latter dominates the delivery in this situation.

Supporting Table 1. Boundary conditions. The point on the surface below the pipette is (x,y,z) = (0,0,0), and each side of the simulation geometry is 10 μm (width and depth equal to 9 μm when delivering to the crest, where the point on the surface below the pipette corresponds to (x,y,z) = (1,1,1) μm in Fig. 2).

	**Boundary condition**
**Electrostatics**	
Top, inside the pipette at z = 10 μm,[1]	$\Psi = \Delta\Psi\times(1-R_0/R_{top})$
x = 10 μm; y = 10 μm; z = 10 μm (outside the pipette)	$\Psi = 0$
All other boundaries,[2]	$\boldsymbol{n}\cdot\nabla\Psi = 0$
**Creeping flow**	
Top, inside the pipette at z = 10 μm	$p = 0$, $(\nabla\mathbf{u}+(\nabla\boldsymbol{u})^{\mathrm{T}})\boldsymbol{n} = \mathbf{0}$
x = 10 μm; y = 10 μm; z = 10 μm (outside the pipette)	$p = 0$, $(\nabla\mathbf{u}+(\nabla\boldsymbol{u})^{\mathrm{T}})\boldsymbol{n} = \mathbf{0}$
Pipette walls	$\boldsymbol{u} = -\mu_{eo}\nabla\Psi$
$x = 0$; $y = 0$	$\boldsymbol{u}\cdot\boldsymbol{n} = 0$, $(\nabla\mathbf{u}+(\nabla\boldsymbol{u})^{\mathrm{T}})\boldsymbol{n} - ((\nabla\mathbf{u}+(\nabla\boldsymbol{u})^{\mathrm{T}})\boldsymbol{n}\cdot\boldsymbol{n})\boldsymbol{n} = \boldsymbol{0}$
All other boundaries	$\boldsymbol{u} = \mathbf{0}$
**Transport of diluted species**	
Top, inside the pipette at z = 10 μm	$c = c_0$
x = 10 μm; y = 10 μm; z = 10 μm (outside the pipette)	$c = c_{flat}$,[3]
All other boundaries	$\boldsymbol{J}\cdot\boldsymbol{n} = \boldsymbol{0}$

[1] R_{top} = 0.50 μm, inner radius of the pipette at z = 10 μm when delivering to the T-tubule.

[2] $\boldsymbol{n}$ = unit vector to the surface boundaries.

[3] c_{flat} is the expression for the concentration profile on a flat surface using Eqs. 1 and 2 in the main text.

Approximate expressions have been used to calculate the boundary conditions for the concentration far from the pipette in order to reduce the simulation volume, similar to our previous work (5). The features on the surface will far from the pipette have less influence on the concentration profile, which will approach the values for a flat surface. The concentration at those distances can thus be set to the values for a flat surface. The voltage at the top of the pipette has also been compensated for by having a truncated pipette (with the length 9.5 μm when delivering to a T-tubule and 8.5 μm when delivering to the crest in-between Z-grooves). No significant change in the simulated concentration was observed for the crest delivery simulations if a truncated pipette length of 9.5 μm was used instead of 8.5 μm. The approximation that $c = c_0$ at the top of the truncated pipette is approximately valid when $\Delta\Psi > 0$, but is less accurate when $\Delta\Psi < 0$. Equations S1 to S3 were solved using linear MUMPS solvers, with a sufficiently fine mesh size to not produce significant changes in the outcome of the simulations when further refining the mesh.

Supporting Figure 3 shows the concentration profile along the y-axis at $x = 0$ when varying one parameter in Table 1 at a time. The concentration profile was normalized using Eq. 3 to the situation that would correspond to the parameter value in Table 1. For example, when varying the radius, R_0, the concentration profile was multiplied with the factor (50 nm)/R_0, and when varying the diffusivity, D, the data was multiplied with the factor $D/(6.7\times10^{-10}\ \mathrm{m^2/s})$.

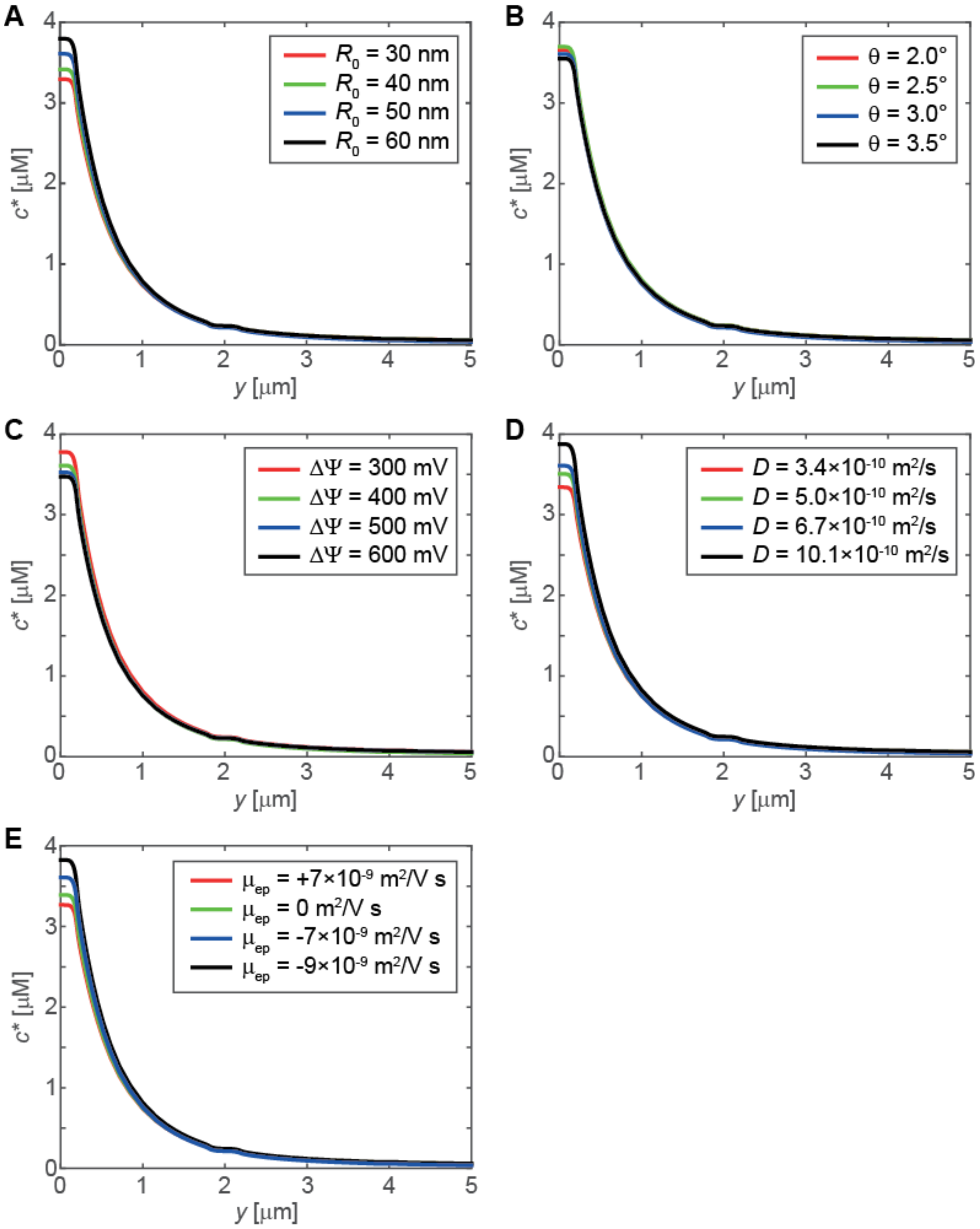

Supporting Figure 3. Various normalized concentration profiles where one parameter at a time has been varied and the other parameters have the value in Table 1. The data is normalized to the parameter value in Table 1 based on the expression in Eq. 3, which for the different situations was done by setting: **(A)** $c^* = c\times((50\text{ nm})/R_0)$, **(B)** $c^* = c\times(\tan(3°)/\tan(\theta))$, **(C)** $c^* = c\times((400\text{ mV})/\Delta\Psi)$, **(D)** $c^* = c\times(D/(6.7\times10^{-10}\text{ m}^2/\text{s}))$ and **(E)** $c^* = c\times((7\times10^{-9}\text{ m}^2/\text{V s})/(\mu_{ep}+14\times10^{-9}\text{ m}^2/\text{V s}))$.

Additional simulations were also performed where the height, *h*, between the pipette and the T-tubule opening was varied under otherwise the same conditions as in Table 1. The obtained concentration profiles, *c*, normalized with the corresponding concentration profiles from Eqs. 1 and 2, c_{flat}, are given in Supporting Figure 4. It can be observed that the concentration at the T-tubule opening increases faster relative to delivery to a flat surface as the pipette approaches the T-tubule opening. When the pipette is far above the Z-groove the concentration profile approaches the values obtained for a flat surface.

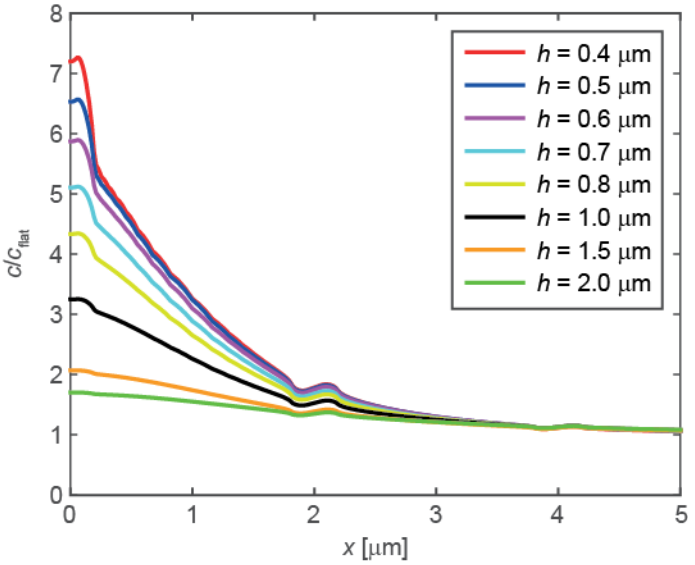

Supporting Figure 4. Concentration profiles at different distances, h, between the tip of the pipette and the T-tubule opening normalized with the theoretical expression in Eqs. 1 and 2 for the concentration profile when delivering to a flat surface. All other parameter values were the same as in Table 1.

Supporting References:

[1] Sato M, O'Gara P, Harding SE, Fuller SJ. (2005) Enhancement of adenoviral gene transfer to adult rat cardiomyocytes in vivo by immobilization and ultrasound treatment of the heart. *Gene Ther*. 12(11):936-41.

[2] Nikolaev VO, Moshkov A, Lyon AR, Miragoli M, Novak P, Paur H, Lohse MJ, Korchev YE, Harding SE, Gorelik J. (2010) Beta2-adrenergic receptor redistribution in heart failure changes cAMP compartmentation. *Science*, 327(5973):1653-7.

[3] Novak P, Li C, Shevchuk AI, Stepanyan R, Caldwell M, Hughes S, Smart TG, Gorelik J, Ostanin VP, Lab MJ, Moss GW, Frolenkov GI, Klenerman D, Korchev YE. (2009) Nanoscale live-cell imaging using hopping probe ion conductance microscopy. *Nat. Methods* 6(4):279-81.

[4] Edelstein A.D, Tsuchida MA, Amodaj N, Pinkard H, Vale RD, Stuurman N. (2014) Advanced methods of microscope control using µManager software. *J. Biol. Methods* 1(2):e10.

[5] Babakinejad B., Jönsson P, López Córdoba A, Actis P, Novak P, Takahashi Y, Shevchuk A, Anand U, Anand P, Drews A, Ferrer-Montiel A, Klenerman D, Korchev YE. (2013) Local delivery of molecules from a nanopipette for quantitative receptor mapping on live cells. *Anal. Chem.* 85(19):9333-42.
