# Supplementary material for: Nanoscale, Voltage-Driven Application of Bioactive Substances onto Cells with Organized Topography
Source: Biophys J. 2016 Jan 5;110(1):141–6. doi: 10.1016/j.bpj.2015.11.017 (PMC4805872; doi:10.1016/j.bpj.2015.11.017)
Supplement: Document S2. Article plus Supporting Material [file mmc2.pdf]

Article

Nanoscale, Voltage-Driven Application of Bioactive Substances onto Cells with Organized Topography

Sophie Schobesberger,[1] Peter Jönsson,[3] Andrey Buzuk,[1] Yuri Korchev,[1] Jennifer Siggers,[2] and Julia Gorelik[1,*]
[1]Department of Medicine and [2]Department of Bioengineering, Imperial College London, London, United Kingdom; and [3]Department of Chemistry, Lund University, Lund, Sweden

ABSTRACT With scanning ion conductance microscopy (SICM), a noncontact scanning probe technique, it is possible both to obtain information about the surface topography of live cells and to apply molecules onto specific nanoscale structures. The technique is therefore widely used to apply chemical compounds and to study the properties of molecules on the surfaces of various cell types. The heart muscle cells, i.e., the cardiomyocytes, possess a highly elaborate, unique surface topography including transverse-tubule (T-tubule) openings leading into a cell internal system that exclusively harbors many proteins necessary for the cell's physiological function. Here, we applied isoproterenol into these surface openings by changing the applied voltage over the SICM nanopipette. To determine the grade of precision of our application we used finite-element simulations to investigate how the concentration profile varies over the cell surface. We first obtained topography scans of the cardiomyocytes using SICM and then determined the electrophoretic mobility of isoproterenol in a high ion solution to be -7×10^{-9} m^2/V s. The simulations showed that the delivery to the T-tubule opening is highly confined to the underlying Z-groove, and especially to the first T-tubule opening, where the concentration is ~6.5 times higher compared to on a flat surface under the same delivery settings. Delivery to the crest, instead of the T-tubule opening, resulted in a much lower concentration, emphasizing the importance of topography in agonist delivery. In conclusion, SICM, unlike other techniques, can reliably deliver precise quantities of compounds to the T-tubules of cardiomyocytes

INTRODUCTION

In cell physiology, increasing attention is paid to the specific location of receptors and proteins on the highly sensitive surface membrane of biological samples like neurons (1), bacilli (2), platelets (3), and heart muscle cells (4,5). The specific location of molecules on the cell surface may underlie differences in their activity in health, and their positioning can change under disease conditions (5). Such differences and changes cannot be studied efficiently with, for example, whole-cell stimulation of receptors (5) or activation of channels (6), but requires a more thorough method of investigation. We have previously investigated the intricately structured beating cells of the heart, the cardiomyocytes. These cells exhibit unique structural features of nanoscale dimension with immense importance to their physiological function. The external topography of cardiomyocytes can be categorized into crests and surface grooves (Z-grooves) in which the transverse-tubule (T-tubule) openings reside and protrude deep into the cell body (7). Due to their small size and their spatial arrangement, these T-tubules generate a highly regulated and very unique environment for the ions needed in excitation-contraction coupling, the process of translating an electrical impulse into the physiological beating response of cardiomyocytes (8). In healthy cardiomyocytes, the T-tubule openings exclusively house a plethora of receptors and ion channels including the $\beta 2$ adrenergic receptors (β_2ARs), the L-type Ca^{2+} channels, and the Na^+/Ca^{2+} exchanger, which are vital for cardiomyocyte function (5). The β_2ARs can be activated by adrenaline or its chemical analog, isoproterenol (9). Scanning ion conductance microscopy (SICM) (10) is a noncontact scanning technique (11), which has often been used to investigate the nanoscale structure of the surface of live cells (3–5). This is achieved by measuring the ion current between an electrode inside the nanopipette and an electrode in the surrounding bath solution (11). The SICM nanopipette can furthermore be used to apply chemical agonists onto different surface structures of the sample with high precision (5). Our group have previously used air pressure to displace the electrolyte solution in the nanopipette for such application (5). However, during the relatively slow air-pressure applications, the nanopipette can be blocked. Hence, our group sought a faster and more reliable method of application. This could be achieved by switching the direction of the electrical current applied over the nanopipette and using this to deliver the agonist (12,13). However, the amplitude (amount of molecules being delivered) and precision of this application method have not been investigated. This knowledge is vital for interpreting the results obtained from delivery

Submitted June 1, 2015, and accepted for publication November 11, 2015.

*Correspondence: j.gorelik@imperial.ac.uk

This is an open access article under the CC BY license (http://creativecommons.org/licenses/by/4.0/).

Sophie Schobesberger and Peter Jönsson contributed equally to this work.

Editor: Mark Cannell.

© 2016 The Authors

0006-3495/16/01/0141/6

http://dx.doi.org/10.1016/j.bpj.2015.11.017

experiments correctly, as well as for future research avenues that SICM could open up. No other technique to our knowledge holds as much promise for the investigation and manipulation of small and precise structures such as, for instance, a single T-tubule. Hence, the aim of this work is to use finite-element simulations to quantify the delivery of isoproterenol to heart cell structures.

MATERIALS AND METHODS

Cardiomyocytes were plated on a dish and perfused with a physiological (pH 7.3) electrolyte solution (144 mM NaCl, 5 mM KCl, and 1 mM $MgCl_2$; see the Supporting Material for details on the preparation of the cells). After acquiring the surface topography with SICM, the nanopipette, prefilled with 50 μM Isoproterenol, was directed over a T-tubule opening. Then the electrical holding potential of −200 mV was changed to +400 mV to expel the chemical agonist by electroosmotic forces. The electroosmotic drift velocity is independent of the charge of the molecules in solution and arises from mobile cations in the electric double layer next to the pipette wall. For the negatively charged glass wall we use a value for the electroosmotic mobility, μ_{eo}, of 1.4×10^{-8} m^2/V s, which approximately corresponds to the value in an electrolyte solution with 150 mM Na^+ (13). This will in turn give rise to a convective liquid flow in the direction of the electric field. However, since isoproterenol is negatively charged, the delivery will be counteracted by an electrophoretic drift component (14). The electrophoretic mobility, μ_{ep}, of isoproterenol was measured in the aforementioned electrolyte solution at 25°C using phase analysis light scattering (PALS), with a NanoBrook ZetaPALS (Brookhaven Instruments, Stockport, United Kingdom). From the Smoluchowski drift-diffusion equation, this resulted in an average value of $-7 \times 10^{-9} \pm 0.2 \times 10^{-9}$ m^2/V s (mean value ± 1 SD).

The molecular flow rate out of the pipette, corresponding to the number of molecules of Isoproterenol leaving the pipette per second, can be shown to be approximately given by (13)

$$Q_{tot} = c_0(\mu_{ep} + \mu_{eo})\pi R_0 \tan(\theta)\Delta\Psi, \quad (1)$$

where c_0 is the concentration of molecules in the bulk of the pipette, R_0 is the inner tip radius of the pipette, θ is the inner half-cone angle, and $\Delta\Psi$ is the voltage drop over the pipette. When the molecules leave the pipette they will be rapidly diluted due to diffusion. It can be shown that for a flat surface, the concentration at distances of $R = (x^2 + y^2 + (z-h)^2)^{0.5} >> R_0$ from the tip of the pipette at $(0,0,z = h)$ approximately varies according to the expression given by

$$c(x,y) = c_0\left(2 - \exp\left(-Q_{tot}\Big/\left(4\pi c_0 D\sqrt{x^2+y^2+(z-h)^2}\right)\right) - \exp\left(-Q_{tot}\Big/\left(4\pi c_0 D\sqrt{x^2+y^2+(z+h)^2}\right)\right)\right), \quad (2)$$

where D is the diffusivity of the molecules and h *is* the distance between the tip of the pipette and the surface. When $c << c_0$, Eq. 2 simplifies to the expression on the flat surface at $z = 0$:

$$c(x,y) \approx c_0 \frac{(\mu_{ep} + \mu_{eo})R_0 \tan(\theta)\Delta\Psi}{D\sqrt{x^2 + y^2 + h^2}}. \quad (3)$$

The concentration thus scales approximately inversely with the distance to the point (0,0) and increases linearly with the radius of the pipette tip and the applied voltage. However, it is not known what the magnitude of the concentration outside the pipette will be, and how the concentration profile will look, when the surface is not flat. To investigate this, we first measured the topography of typical cardiomyocyte surface structures using SICM. From the obtained surface scans (Fig. 1), the number of T-tubule openings and Z-grooves on the cardiomyocytes was determined and averaged to obtain the necessary parameters for the simulation geometry (Fig. 2). Finite-element simulations using the program COMSOL Multiphysics 5.0 (COMSOL, Burlington, MA) were performed to estimate the delivered amount of Isoproterenol, using the parameter values in Table 1. Additional information about the details of the numerical simulations and on the mathematical boundary conditions can be found in the Supporting Material.

RESULTS

Under experimental conditions the β_2AR-dependent second messenger signal in the cardiomyocytes only changed when isoproterenol was applied into the T-tubule opening on the cell surface and not on the crest area between the Z-grooves, as can be seen in Fig. 1, *B* and *C* (a detailed description of the SICM and the β_2AR-dependent second messenger signal measurements are given in the Supporting Material). To estimate the amount of isoproterenol delivered, we used finite-element simulations to establish the approximate amount of the molecule isoproterenol being delivered from a nanopipette to underlying cardiomyocyte structures and determined the delivered concentration profile over the highly structured heart cell surfaces. To define the parameters of the model, we determined a representative model of the cell surface, the pipette dimensions, and the ligand's electrophoretic mobility.

The diameter of the T-tubule opening at the primary application site was measured to be ~400 nm. Based on the aforementioned values, a representative model of healthy cardiomyocytes was constructed from the SICM images (Figs. 1 *A* and 2). The simulation geometry consists of a 10 μm^3 cube with parts subtracted to construct

1) grooves 0.4 μm wide and 1 μm high along the *y*-direction, spaced 2 μm apart;
2) grooves 0.4 μm wide and 0.25 μm high in the *x*-direction, spaced 2 μm apart; and
3) a pipette with an inner tip radius of 50 nm, an outer radius of 100 nm, and an inner half-cone angle of 3°.

The simulated concentration outside the pipette when delivering with a voltage of 400 mV is shown in Fig. 3 together with the situation when delivering with $\Delta\Psi = -200$ mV. For the positive delivery voltage 7×10^5 molecules/s of isoproterenol are leaving the pipette, which is in good agreement with the predicted value from Eq. 1. Thus, approximately the same amount of isoproterenol is leaving the pipette when delivering into the Z-groove as when delivering to a flat surface, which is the case as long as the flow resistance outside the pipette is smaller than the flow resistance in the interior of the pipette (12). However, the concentration profiles outside the pipette will be different for the two cases, as discussed below. There is

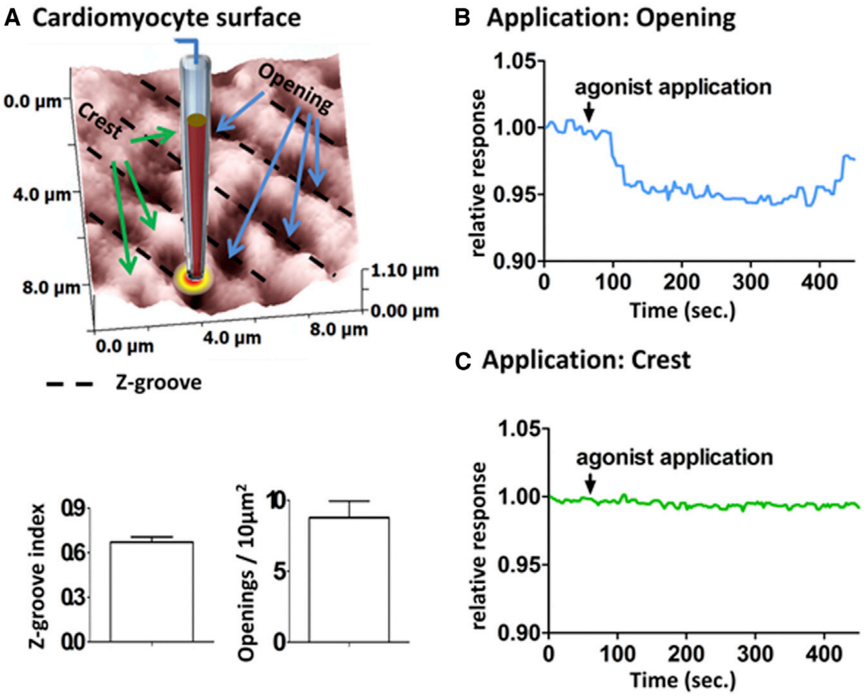

FIGURE 1 (*A*) (*Upper*) A 10 × 10 μm^2 SICM surface scan, schematically showing isoproterenol application into an opening on the surface of a cardiomyocyte. Z-grooves are indicated by black dotted lines, openings by blue arrows, and crests by green arrows. (*Lower*) Summary of the respective Z-groove index, an indicator of how many Z-grooves are present, which is defined by determining the length of all Z-grooves and dividing this value by the maximally extrapolated length possible on the 10 × 10 μm^2 scan (7) (*lower left*), and the number of openings on the 10 × 10 μm^2 area (*lower right*) (N = 10). (*B* and *C*) Graphs showing the cell internal β_2AR-dependent second messenger response to agonist application into the T-tubule opening (*B*) and onto the crest of the cell surface (*C*), obtained by moving the pipette to the respective coordinates on the computer-generated SICM scan. To see this figure in color, go online.

also a flow of molecules out of the pipette due to diffusion when applying $\Delta\Psi = -200$ mV. This value is ~5 × 10^4 molecules/s, which is an order of magnitude lower than the value at $\Delta\Psi = 400$ mV, but is not zero. It can therefore be advisable to withdraw the pipette from the sample between delivery time points to avoid excessive delivery, and thus stimulus, of the cardiomyocytes. Note also, that the concentration in the bulk of the pipette is 50 μM, but that the color scale in Fig. 3 is between 0 and 5 μM to better visualize the concentration profile outside the pipette. Details about the finite-element simulations are given in the Supporting Material.

The molecular flow of isoproterenol is initially guided along the groove at $x = 0$ in the y-direction. The concentration along the lower edge at $x = 0$ and the lower edge at $y = 0$ is shown in Fig. 3 *D*, where the concentration is also compared to the corresponding value for a flat surface, $h = 0.5$ μm below the pipette, using the theoretical expressions in Eqs. 1 and 2. The distance is given as the value of y for the edge at $x = 0$ and as the value of x for the edge at $y = 0$.

The concentration at the T-tubule opening at $(x, y, z) = (0, 0, 0)$ is ~3.5 μM, which is ~6.5 times higher than the concentration for a flat surface at the same distance to the pipette. The reason for this behavior is that the molecules are initially limited to diffusion along the length of the

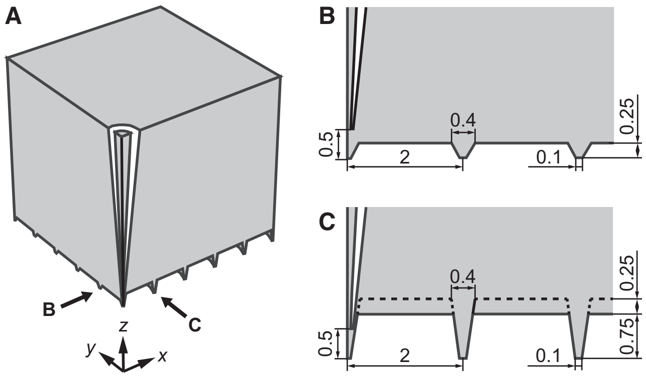

FIGURE 2 The geometry used in the finite-element simulations of a cardiomyocyte. (*A*) The simulation geometry consists of a cube with a side length of 10 μm, in which parts have been subtracted to make up the Z-grooves and T-tubule openings as well as the pipette. (*B*) A zoom-in of the yz-plane at $x = 0$ together with dimensions of the simulation geometry. (*C*) A zoom-in of the xz-plane at $y = 0$ together with dimensions of the simulation geometry. The pipette has an inner tip radius of 50 nm, an outer tip radius of 100 nm, and an inner half-cone angle of 3°. All distances are given in micrometers.

TABLE 1 Parameters and Values for Modeling the Delivery of Isoproterenol

Name	Description	Value
R_0	inner tip radius	50 nm
R_1	outer tip radius	100 nm
θ	inner half-cone angle	3°
h	pipette-surface distance	500 nm
D	diffusivity of ISO (15)	6.7×10^{-10} m^2/s
μ_{ep}	electrophoretic mobility of ISO	-7×10^{-9} m^2/V s
μ_{eo}	electroosmotic mobility in the pipette (13)	1.4×10^{-8} m^2/V s
c_0	concentration of ISO inside the pipette	50 μM
$\Delta\Psi$	applied voltage over the pipette	400 mV

ISO, isoproterenol.

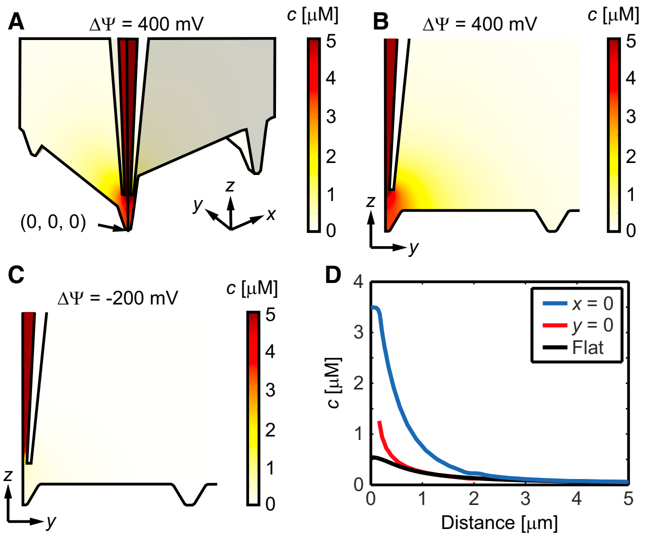

FIGURE 3 Delivery of isoproterenol to a T-tubule opening. (*A*) 3D image showing the simulated concentration of isoproterenol when delivering with $\Delta\Psi = 400$ mV. The concentration in the bulk of the pipette is 50 μM. (*B* and *C*) 2D zoom-in at the plane $x = 0$ showing the concentration distribution at $\Delta\Psi = 400$ mV and -200 mV, respectively. (*D*) Line profiles of the concentration at $\Delta\Psi = 400$ mV along the lower edge at $x = 0$ (*blue*) and $y = 0$ (*red*) as a function of the distances *y* and *x*, respectively, from the point $(x, y, z) = (0, 0, 0)$. The black line is the corresponding value for a flat surface at $z = 0$ using Eqs. 1 and 2. To see this figure in color, go online.

Z-groove, which results in a slower decrease of the concentration in this direction. At larger distances, the molecules start to diffuse in all directions again and the concentration approaches that of a flat surface. In fact, the concentration at the T-tubule opening in the second groove at $(x, y, z) = (2\ \mu\text{m}, 0, 0)$ is only ~10% different from the value for a flat surface. This value is ~30 times lower than the concentration at the T-tubule opening at $(x, y, z) = (0, 0, 0)$. The concentration for the second T-tubule opening in the groove at $(x, y, z) = (0, 2\ \mu\text{m}, 0)$ is higher due to the guiding effect of the groove, but it is still ~15 times lower compared to the concentration over the first opening (at $x = y = 0$), indicating that the delivery is mainly limited to the T-tubule opening beneath the pipette. Another possibility is that the Z-groove focuses the flow out of the pipette, and that this effect is causing the increase in concentration at the underlying T-tubule opening. However, we have previously observed that the concentration profile outside nanopipettes, at a distance larger than one pipette radius from the tip, is generally dominated by diffusion and that the convective term here, as a first approximation, can be neglected (13). This was found also to be true for delivery to a Z-groove, since setting the convective term equal to zero outside the pipette only resulted in a 20% drop in the concentration at the T-tubule opening, significantly less than the 6.5 times decrease when delivering to a flat surface.

When instead delivering to the crest between the openings ($x = y = 1\ \mu$m in Fig. 2), $h = 500$ nm above the surface, the concentration in the nearest T-tubule is ~20 times lower compared to when delivering directly to the T-tubule (Fig. 4). This is well in line with the observation that there is a response only if delivering agonist to the T-tubule opening and not to the crest, as shown in Fig. 1, *B* and *C*.

The concentration profile will change for different values of the parameters in Table 1, but will approximately scale with $\Delta\Psi$, R_0 (assuming $R_1 = 2R_0$), θ, D, and μ_{ep} according to the expression in Eq. 3 when $c << c_0$ (see Fig. S3). For example, for a pipette with a 20% larger radius the concentration will be 20% larger if all other parameters are kept constant and, similarly, the concentration will scale roughly linearly with the applied voltage under the condition that $c << c_0$. Simulations were also made where the distance between the tip of the pipette and the T-tubule opening, h, was varied, but otherwise under the same conditions as summarized in Table 1. The results from these simulations are shown in Fig. S4, indicating that the difference in concentration at the T-tubule opening, compared to delivery onto a flat surface, is largest when the pipette is close to the T-tubule opening. When the pipette retracts further from the Z-groove, the concentration profile approaches that for delivery to a flat surface. A COMSOL Multiphysics 5.0 file of the simulations described in this work is included in the Supporting Materials.

The charge distribution from an electrical double layer close to the walls of the pipette has so far been neglected in

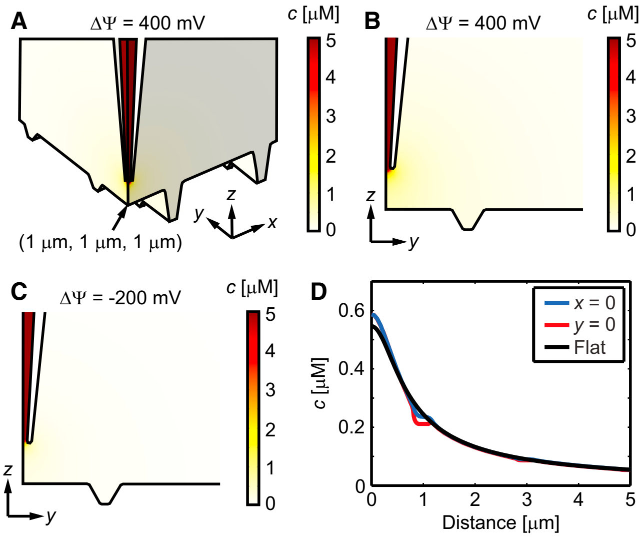

FIGURE 4 Delivery of isoproterenol to the crest between T-tubule openings. (*A*) 3D image showing the concentration of isoproterenol when delivering with $\Delta\Psi = 400$ mV. The concentration in the bulk of the pipette is 50 μM. (*B* and *C*) 2D zoom-in at the plane $x = 1\ \mu$m showing the concentration distribution at $\Delta\Psi = 400$ mV and $\Delta\Psi = -200$ mV, respectively. (*D*) Line profiles of the concentration along the lower edge at $x = 1\ \mu$m (*blue*) and $y = 1\ \mu$m (*red*) as a function of the distances *y* and *x*, respectively, from the point $(x, y, z) = (1, 1, 1)\ \mu$m. The black line is the corresponding value for a flat surface using Eqs. 1 and 2 (relative to the point (1, 1, 1) μm). To see this figure in color, go online.

the simulations of the electric field. However, for small nanopipettes this can give rise to a significant change in both the electric field and the electroosmotic flow through the pipette (17). Additional simulations were therefore made to investigate this effect on the current system, where the concentration of Na^+ and Cl^- in the nanopipette was simulated and used as the charge distribution for the electric field simulations in the nanopipette. COMSOL Multiphysics was again used for this following the procedure outlined by Ivanov et al. (17). The dimensions of the pipette are given in Table 1 and the size of the simulation geometry was scaled up by a factor of 4 to take into account the larger pipette size compared to the nanopipettes used by Ivanov et al. Only the situation with a pipette far from the underlying surface was investigated, which was considered adequate to obtain an estimate of how the electric field at the tip of the pipette and the total osmotic flow are affected by the electrical double layer. For the pipette walls, a surface charge of $\sigma = -18$ mC/m^2 was assumed (corresponding to a ζ potential of $\zeta = -20$ mV (17)), which gives an electroosmotic mobility of 1.4×10^{-8} m^2/V s. The concentration of Na^+ and Cl^- was set to 150 mM, and the diffusivities of Na^+ and Cl^- used were $D_{Na^+} = 1.33 \times 10^{-9}$ m^2/s and $D_{Cl^-} = 2.03 \times 10^{-9}$ m^2/s, respectively (18). Under these conditions, only modest differences in the electroosmotic flow out of the pipette were found. The total electroosmotic flow rate out of the pipette deviated by $<2\%$ when including the effect of the electrical double layer, and the deviation in the electric field in the z-direction, along the axis of the pipette, was $<10\%$. Thus, for the current situation, where the inner tip radius of the nanopipette is ~60 times larger than the Debye length at the salt concentration used, the effect of the electrical double layer on the electric field is only minor. However, this effect might be more pronounced for smaller pipettes, or when using low salt concentrations.

Our results show that delivery to a single opening on the cell surface can be made repeatedly and reliably, but also that the effect of the topography on the delivery can be significant. This is of special importance in the unique case of cardiomyocytes, as their structures hold the key to their proper physiological function.

DISCUSSION

An increasing number of investigations highlight the importance of compartment-specific signaling of this cell type, and investigation and manipulation of single compartments will become crucial for fully understanding their physiological and pathophysiological regulation. So far, to our knowledge, SICM is the only method that enables us to perform noninvasive experiments that are as localized as a single T-tubule opening. The analytical verification of the application method presented here encourages a range of further local application experiments. The technique allows for delivery of essentially any compound to multiple structured cell types with a precision determined by the pipette dimensions and the potential compound charge. In the case of cardiology, this concept could be applied to delivering fluorescent dyes and investigating diffusion dynamics within the T-tubule network, delivering universal and specific agonists as well as ions, for example, Ca^{2+} to monitor induced Ca^{2+} release. The concept could also be expanded to local application after membrane sealing by the nanopipette, which would avoid diffusion of the agonist into the extracellular space and lead to higher concentrations at the structure. Therefore, we present a universal approach to quantify local application and to adjust such parameters as precision, speed, and ultimately concentration through modulating the current and pipette structure, which promises to yield important data in cardiology and beyond.

CONCLUSION

Even though the simulations presented here were for the specific case of isoproterenol delivery to cardiomyocytes, we propose that they can also be applied and made useful for other cell types and agonist delivery applications, as they allow the quantification, and possible optimization, of application processes that deal with nanoscale structures that evade experimental determination.

SUPPORTING MATERIAL

Supporting Materials and Methods, four figures, and one table are available at http://www.biophysj.org/biophysj/supplemental/S0006-3495(15)01174-1.

SUPPORTING REFERENCES

References (19,20) appear in the Supporting Material.

AUTHOR CONTRIBUTIONS

Research design was carried out by S.S., P.J., J.S., and J.G.; research was performed by S.S. and P.J.; graphics were generated by S.S., P.J., A.B., and Y.K.; analytic tools were contributed by P.J., Y.K., and J.G.; data were analyzed by S.S., P.J., and A.B.; and the article was written by S.S., P.J., A.B., J.S., and J.G.

ACKNOWLEDGMENTS

Dan Clarke, from the company Brookhaven Instruments, in the United Kingdom, is gratefully acknowledged for providing isoproterenol electric mobility measurements and outstanding, problem-solving customer service.

This research was supported by the British Heart Foundation (BHF). P.J. was supported by grants from the Swedish Research Council (623-2014-6387 and 621-2014-3907).

REFERENCES

1. Takahashi, Y., A. I. Shevchuk, …, T. Matsue. 2012. Topographical and electrochemical nanoscale imaging of living cells using

voltage-switching mode scanning electrochemical microscopy. *Proc. Natl. Acad. Sci. USA.* 109:11540–11545.

2. Shevchuk, A. I., G. I. Frolenkov, …, Y. E. Korchev. 2006. Imaging proteins in membranes of living cells by high-resolution scanning ion conductance microscopy. *Angew. Chem. Int. Ed. Engl.* 45:2212–2216.
3. Liu, X., Y. Li, …, Y. Zhang. 2015. Use of non-contact hopping probe ion conductance microscopy to investigate dynamic morphology of live platelets. *Platelets.* 26:480–485.
4. Lorin, C., M. Gueffier, …, S. Sebille. 2013. Ultrastructural and functional alterations of EC coupling elements in *mdx* cardiomyocytes: an analysis from membrane surface to depth. *Cell Biochem. Biophys.* 66:723–736.
5. Nikolaev, V. O., A. Moshkov, …, J. Gorelik. 2010. β2-adrenergic receptor redistribution in heart failure changes cAMP compartmentation. *Science.* 327:1653–1657.
6. Bhargava, A., X. Lin, …, J. Gorelik. 2013. Super-resolution scanning patch clamp reveals clustering of functional ion channels in adult ventricular myocyte. *Circ. Res.* 112:1112–1120.
7. Gorelik, J., L. Q. Yang, …, S. E. Harding. 2006. A novel Z-groove index characterizing myocardial surface structure. *Cardiovasc. Res.* 72:422–429.
8. Hatano, A., J. Okada, …, S. Sugiura. 2012. Critical role of cardiac t-tubule system for the maintenance of contractile function revealed by a 3D integrated model of cardiomyocytes. *J. Biomech.* 45:815–823.
9. Minneman, K. P., L. R. Hegstrand, and P. B. Molinoff. 1979. The pharmacological specificity of β-1 and β-2 adrenergic receptors in rat heart and lung in vitro. *Mol. Pharmacol.* 16:21–33.
10. Hansma, P. K., B. Drake, …, C. B. Prater. 1989. The scanning ion-conductance microscope. *Science.* 243:641–643.
11. Novak, P., C. Li, …, Y. E. Korchev. 2009. Nanoscale live-cell imaging using hopping probe ion conductance microscopy. *Nat. Methods.* 6:279–281.
12. Ying, L., A. Bruckbauer, …, D. Klenerman. 2005. The scanned nanopipette: a new tool for high resolution bioimaging and controlled deposition of biomolecules. *Phys. Chem. Chem. Phys.* 7:2859–2866.
13. Babakinejad, B., P. Jönsson, …, Y. E. Korchev. 2013. Local delivery of molecules from a nanopipette for quantitative receptor mapping on live cells. *Anal. Chem.* 85:9333–9342.
14. Nixon, G. I., and G. W. Slater. 1996. Entropic trapping and electrophoretic drift of a polyelectrolyte down a channel with a periodically oscillating width. *Phys. Rev. E Stat. Phys. Plasmas Fluids Relat. Interdiscip. Topics.* 53:4969–4980.
15. Venter, J. C. 1978. Cardiac sites of catecholamine action: diffusion models for soluble and immobilized catecholamine action on isolated cat papillary muscles. *Mol. Pharmacol.* 14:562–574.
17. Ivanov, A. P., P. Actis, …, J. B. Edel. 2015. On-demand delivery of single DNA molecules using nanopipets. *ACS Nano.* 9:3587–3595.
18. Vanýsek, P. 2015. Ionic conductivity and diffusion at infinite dilution. *In* CRC Handbook of Chemistry and Physics, 96th ed. (Internet Version 2015). W. M. Haynes, editor. CRC Press/Taylor and Francis, Boca Raton, FL.
19. Sato, M., P. O'Gara, …, S. J. Fuller. 2005. Enhancement of adenoviral gene transfer to adult rat cardiomyocytes in vivo by immobilization and ultrasound treatment of the heart. *Gene Ther.* 12:936–941.
20. Edelstein, A. D., M. A. Tsuchida, …, N. Stuurman. 2014. Advanced methods of microscope control using μManager software. *J. Biol. Methods.* 1:e10.

Biophysical Journal, Volume *110*

Supplemental Information

Nanoscale, Voltage-Driven Application of Bioactive Substances onto Cells with Organized Topography

Sophie Schobesberger, Peter Jönsson, Andrey Buzuk, Yuri Korchev, Jennifer Siggers, and Julia Gorelik

Precise, nanoscale, voltage-driven application of bio-active substances onto cardiomyocyte surface with organized topography

Sophie Schobesberger‡¤, Peter Jönsson‡†, Andrey Buzuk¤, Yuri Korchev¤, Jennifer Siggers¶, Julia Gorelik¤*

¤Department of Medicine, Imperial College London, London, United Kingdom

¶Department of Bioengineering, Imperial College London, London, United Kingdom

† Department of Chemistry, Lund University, Lund, Sweden

Author Contributions

‡These authors contributed equally.

Correspondence to: Julia Gorelik, PhD (e-mail: j.gorelik@imperial.ac.uk, Tel: +44 (0)20 7594 2736, Fax: 44 (0)20 7594 3653), Department of Cardiac Medicine, National Heart and Lung Institute, Imperial College, ICTEM 4th floor, London W12 0NN, UK

Supporting Material:

Contents

1. Research Animals 1

2. Cardiomyocyte cell isolation and culture 1

3. Cardiomyocyte transfection with a FRET biosensor to detect the cyclic nucleotide cAMP 2

4. Determination of cell surface structures using Scanning Ion Conductance Microscopy 2

5. Measurements of the β adrenergic receptor response using FRET 2

6. Measurements of the electrophoretic mobility of Isoproterenol 3

1. Research Animals

All procedures were carried out in compliance with the standards for the care and use of animal subjects as stated in the Guide of the Care and Use of Laboratory Animals (NIH publication No. 85–23, revised 1996) and the requirements of the UK Home Office (ASPA1986 Amendments Regulations 2012) incorporating the EU directive *2010/63/EU*. Protocols were approved by the Animal Care and Use Committee of Imperial College London.

2. Cardiomyocyte cell isolation and culture

Cardiomyocytes were obtained from the left ventricle of excised, adult Sprague-Dawley rat hearts via Langendorff perfusion and enzymatic digestion as described previously (1). For cell culture glass bottom dishes (MatTeK corporation, Ashland, USA) were coated with laminin and isolated cardiomyocytes were plated on the dishes and incubated at 5% CO_2 in modified M199 (Invitrogen, UK) culture medium containing per 500 mL bovine serum

albumin (0.5 g/L), creatine (5 mM/L), taurine (5 mM/L), L-ascorbic acid (100 mM/L), carnitine (2 mM), and penicillin/streptomycin (100 mM/L).

3. Cardiomyocyte transfection with a FRET biosensor to detect the cyclic nucleotide cAMP

To detect the cyclic nucleotide cAMP a Förster Resonance Energy Transfer (FRET) biosensor was used. The cAMP sensor Epac2-camps is made of the cAMP-binding domain of the Epac protein together with the donor fluorophore Cyan Fluorescent Protein (CFP) and the acceptor fluorophore Yellow Fluorescent Protein (YFP) attached at either end of the binding domain. Upon binding of cAMP the sensor undergoes a conformational change which brings the acceptor fluorophore apart from the donor fluorophore and the FRET energy exchange drops, which is recorded as a decrease in the fluorescent signal ratio of YFP to CFP. Cells were infected with the adeno-associated virus containing the Epac2-camps sensor and cultured for 48h as described previously (2).

4. Determination of cell surface structures using Scanning Ion Conductance Microscopy

For the visualization of the surface membrane topography of cardiomyocytes the Scanning Ion Conductance Microscope (SICM) in the “hopping” mode was used with nanopipettes of ~100 MΩ resistance as sensitive probes, as described previously (3). In short the SICM allows for a three-dimensional nanoscale topographic layout of the surface structure of live samples, such as T-tubules and crests on cardiomyocytes, to be obtained. This is achieved by measuring the ion current between a reference electrode inside the electrolyte-filled nanopipette, which serves as a nanoprobe, and a ground electrode, which is positioned in the sample dish; the feedback control mechanism keeps the distance between the pipette and the sample constant while the nanoprobe is scanning the sample. The displacement of the pipette is recorded and forms a three-dimensional image. The physiological electrolyte solution (pH 7.3) contained NaCl (144 mM), KCl (5 mM), HEPES (10 mM) and $MgCl_2$ (1 mM).

5. Measurements of the β adrenergic receptor response using FRET

All experiments were performed at room temperature (20-22°C) with the cardiomyocytes expressing the Epac2-camps FRET sensor being pre-incubated with CGP20712A (100 nM), a β_1AR receptor blocker. Topographical images (10×10 µm) were obtained by SICM to visualise the cell surface structure and distinguish between the T-tubule openings and the crest structures, in order to detect local β_2AR dependent cAMP FRET signals. β_2ARs were then stimulated locally via the SICM nanopipette being positioned either over a T-tubule opening or over a crest area. This was done by placing the pipette at defined coordinates according to the previously obtained surface scan, turning off the automatic feedback control that keeps the nanopipette at a constant distance (2500 nm when scanning cardiomyocytes) and lowering down the nanopipette until it is only 500 nm from the chosen surface structure. After establishing a baseline signal for 100 s, Isoproterenol (50 µM) was applied through the nanopipette by changing the electrical potential from -200 mV to 400 mV. The FRET sensor

was excited at 436 nm and the emission, detected with 535/40 nm and 480/30 nm filters, was recorded in two images simultaneously every 5 s with a Hamamatsu ORCA ER camera and the Micro-Manager 1.4 programme (4). The imaging system necessary for this was built around a Nikon TE2000 microscope and has been described previously (2). The schematic of the experiment is shown in **Supporting Figure 1**.

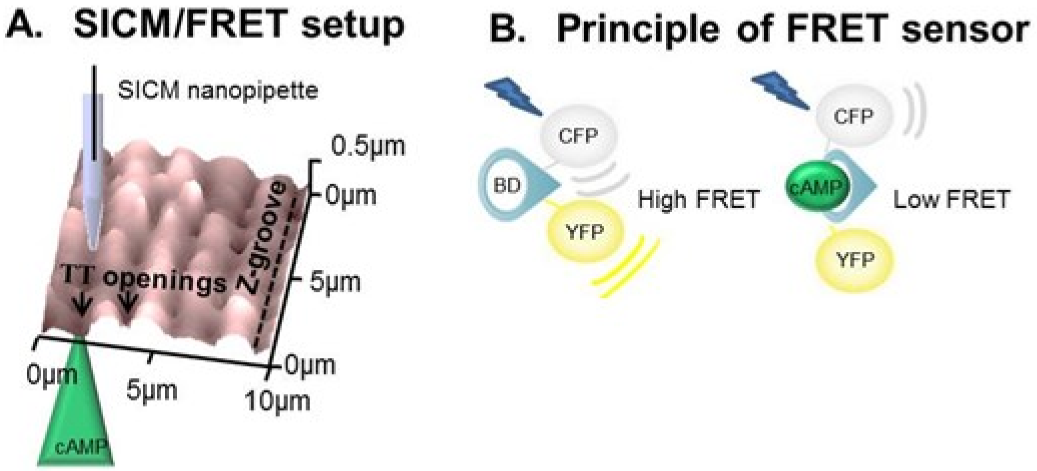

Supporting Figure 1. (A) Schematic of local β_2AR stimulation through a SICM pipette onto the surface of a healthy cardiomyocyte after using SICM to map the topography. **(B)** The principle of cAMP level measurements with the Epac2-camps FRET sensor; once cAMP binds the binding domain (BD) of the Epac protein a change in conformation of the FRET sensor will lead to a decrease in the FRET signal.

6. Measurements of the electrophoretic mobility of Isoproterenol

Measuring electrophoretic mobility can be achieved through Electrophoretic Light Scattering (ELS) techniques, which are based on the fact that moving particles which are hit by laser light will scatter the light according to their size and velocity due to, for example, Brownian motion or motion elicited by electrical fields. The scattered laser light can be detected and compared to a reference laser beam in order to determine the shift in the laser light frequency and with it the properties of the particles. Phase Analysis Light Scattering (PALS) is an advancement of the conventional ELS techniques and uses pulsatile laser beams and determines the motion and size of particles by the thereby generated phase shift of the detected light. By applying pulses only onto small fields PALS does not generate problematic alterations in temperature and does not destroy or change the properties of the sample. The NanoBrook machine used to carry out the PALS measurements furthermore facilitates sample testing in high salt buffer solutions with high conductance as was done here for Isoproterenol. Measurements of the electrophoretic mobility of Isoproterenol in physiological buffer (pH 7.3) containing NaCl (144 mM), KCl (5 mM), HEPES (10 mM) and $MgCl_2$ (1 mM) were conducted by Dr. Dan Clarke, on behalf of the company Brookhaven Instruments, UK. For the measurements the sample was analysed in 5 consecutive runs using a NanoBrook ZetaPALS machine (model: NanoBrook Omni, Brookhaven Instruments Corporation, UK) and phase analysis light scattering (PALS). The correlation-function of the phase shift from which the electrophoretic mobility was determined via the Smoluchowski drift-diffusion equation is shown below in Supporting Figure 2.

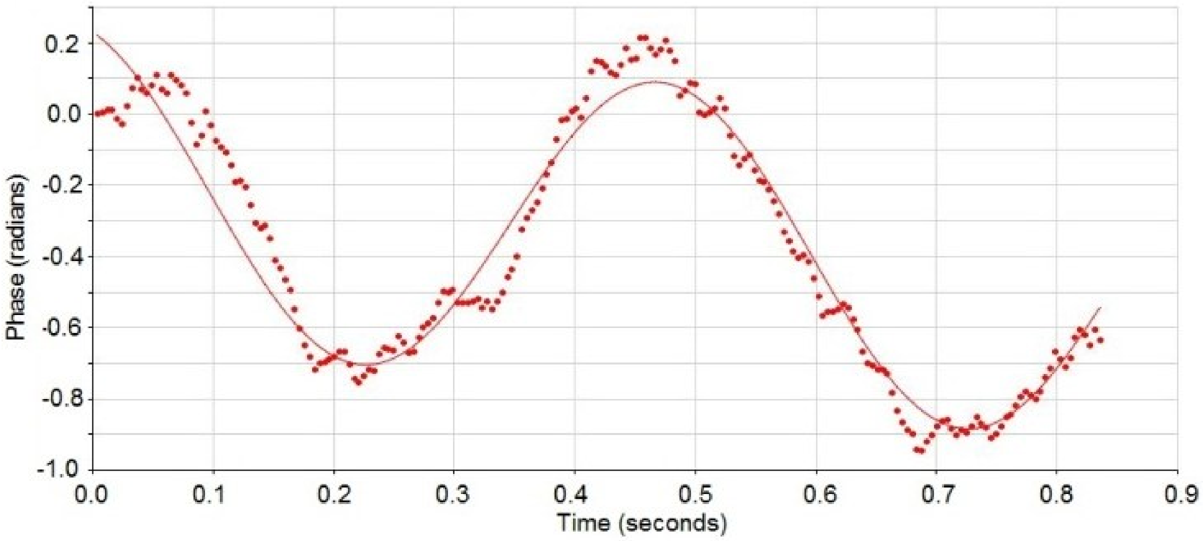

Supporting Figure 2. Correlation-function used to determine the electrophoretic mobility of Isoproterenol in physiological buffer using PALS.

7. Details regarding the numerical simulations

The program COMSOL Multiphysics® 5.0 (COMSOL, Inc.) was used to solve for the concentration of Isoproterenol delivered from the pipette due to a voltage drop, $\Delta\Psi$, applied over the pipette. The time it takes to reach steady state is much faster than the time scales normally used for the delivery and stationary equations can therefore be used. The following three sets of equations were solved:

(Electrostatics) $$\nabla^2\Psi = 0 \tag{S1}$$

(Creeping flow) $$-\nabla p + \eta\nabla^2\boldsymbol{u} = 0 \tag{S2a}$$

$$\nabla\cdot\boldsymbol{u} = 0 \tag{S2b}$$

(Transport of diluted species) $$\nabla\cdot\boldsymbol{J} = 0 \tag{S3a}$$

$$\boldsymbol{J} = -D\nabla c + c\left(\boldsymbol{u} - \mu_{\mathrm{ep}}\nabla\Psi\right) \tag{S3b}$$

where Ψ is the electric potential, p the hydrostatic pressure, $\boldsymbol{u}$ the liquid flow vector, η (= 1 mPa s) the viscosity of the liquid and $\boldsymbol{J}$ the molecular flux of Isoproterenol. Equation S1 gives the electric field in the pipette, and is solved for first. Next, Eq. S2 is solved to determine the electroosmotic flow in the system, where the determined electric field $\boldsymbol{E} = -\nabla\Psi$ is used as input value. The concentration of Isoproterenol is finally determined by solving Eq. S3 with the already simulated values of $\boldsymbol{u}$ and Ψ as input values to determine the amount of Isoproterenol delivered. The boundary conditions used in the simulations are given in Supporting Table 1 with the parameter values in Table 1 in the main text. Note that the electrophoretic and electroosmotic mobility has opposite signs, and that the latter dominates the delivery in this situation.

Supporting Table 1. Boundary conditions. The point on the surface below the pipette is $(x,y,z) = (0,0,0)$, and each side of the simulation geometry is 10 µm (width and depth equal to 9 µm when delivering to the crest, where the point on the surface below the pipette corresponds to $(x,y,z) = (1,1,1)$ µm in Fig. 2).

	**Boundary condition**
**Electrostatics**	
Top, inside the pipette at z = 10 µm,[1]	$\Psi = \Delta\Psi\times(1-R_0/R_{top})$
x = 10 µm; y = 10 µm; z = 10 µm (outside the pipette)	$\Psi = 0$
All other boundaries,[2]	$\boldsymbol{n}\cdot\nabla\Psi = 0$
**Creeping flow**	
Top, inside the pipette at z = 10 µm	$p = 0$, $(\nabla\mathbf{u}+(\nabla\boldsymbol{u})^{T})\boldsymbol{n} = \mathbf{0}$
x = 10 µm; y = 10 µm; z = 10 µm (outside the pipette)	$p = 0$, $(\nabla\mathbf{u}+(\nabla\boldsymbol{u})^{T})\boldsymbol{n} = \mathbf{0}$
Pipette walls	$\boldsymbol{u} = -\mu_{eo}\nabla\Psi$
$x = 0$; $y = 0$	$\boldsymbol{u}\cdot\boldsymbol{n} = 0$, $(\nabla\mathbf{u}+(\nabla\boldsymbol{u})^{T})\boldsymbol{n} - ((\nabla\mathbf{u}+(\nabla\boldsymbol{u})^{T})\boldsymbol{n}\cdot\boldsymbol{n})\boldsymbol{n} = \boldsymbol{0}$
All other boundaries	$\boldsymbol{u} = \mathbf{0}$
**Transport of diluted species**	
Top, inside the pipette at z = 10 µm	$c = c_0$
x = 10 µm; y = 10 µm; z = 10 µm (outside the pipette)	$c = c_{flat}$,[3]
All other boundaries	$\boldsymbol{J}\cdot\boldsymbol{n} = \boldsymbol{0}$

[1] R_{top} = 0.50 µm, inner radius of the pipette at z = 10 µm when delivering to the T-tubule.

[2] $\boldsymbol{n}$ = unit vector to the surface boundaries.

[3] c_{flat} is the expression for the concentration profile on a flat surface using Eqs. 1 and 2 in the main text.

Approximate expressions have been used to calculate the boundary conditions for the concentration far from the pipette in order to reduce the simulation volume, similar to our previous work (5). The features on the surface will far from the pipette have less influence on the concentration profile, which will approach the values for a flat surface. The concentration at those distances can thus be set to the values for a flat surface. The voltage at the top of the pipette has also been compensated for by having a truncated pipette (with the length 9.5 µm when delivering to a T-tubule and 8.5 µm when delivering to the crest in-between Z-grooves). No significant change in the simulated concentration was observed for the crest delivery simulations if a truncated pipette length of 9.5 µm was used instead of 8.5 µm. The approximation that $c = c_0$ at the top of the truncated pipette is approximately valid when $\Delta\Psi > 0$, but is less accurate when $\Delta\Psi < 0$. Equations S1 to S3 were solved using linear MUMPS solvers, with a sufficiently fine mesh size to not produce significant changes in the outcome of the simulations when further refining the mesh.

Supporting Figure 3 shows the concentration profile along the y-axis at $x = 0$ when varying one parameter in Table 1 at a time. The concentration profile was normalized using Eq. 3 to the situation that would correspond to the parameter value in Table 1. For example, when varying the radius, R_0, the concentration profile was multiplied with the factor (50 nm)/R_0, and when varying the diffusivity, D, the data was multiplied with the factor $D/(6.7\times10^{-10}\ \text{m}^2/\text{s})$.

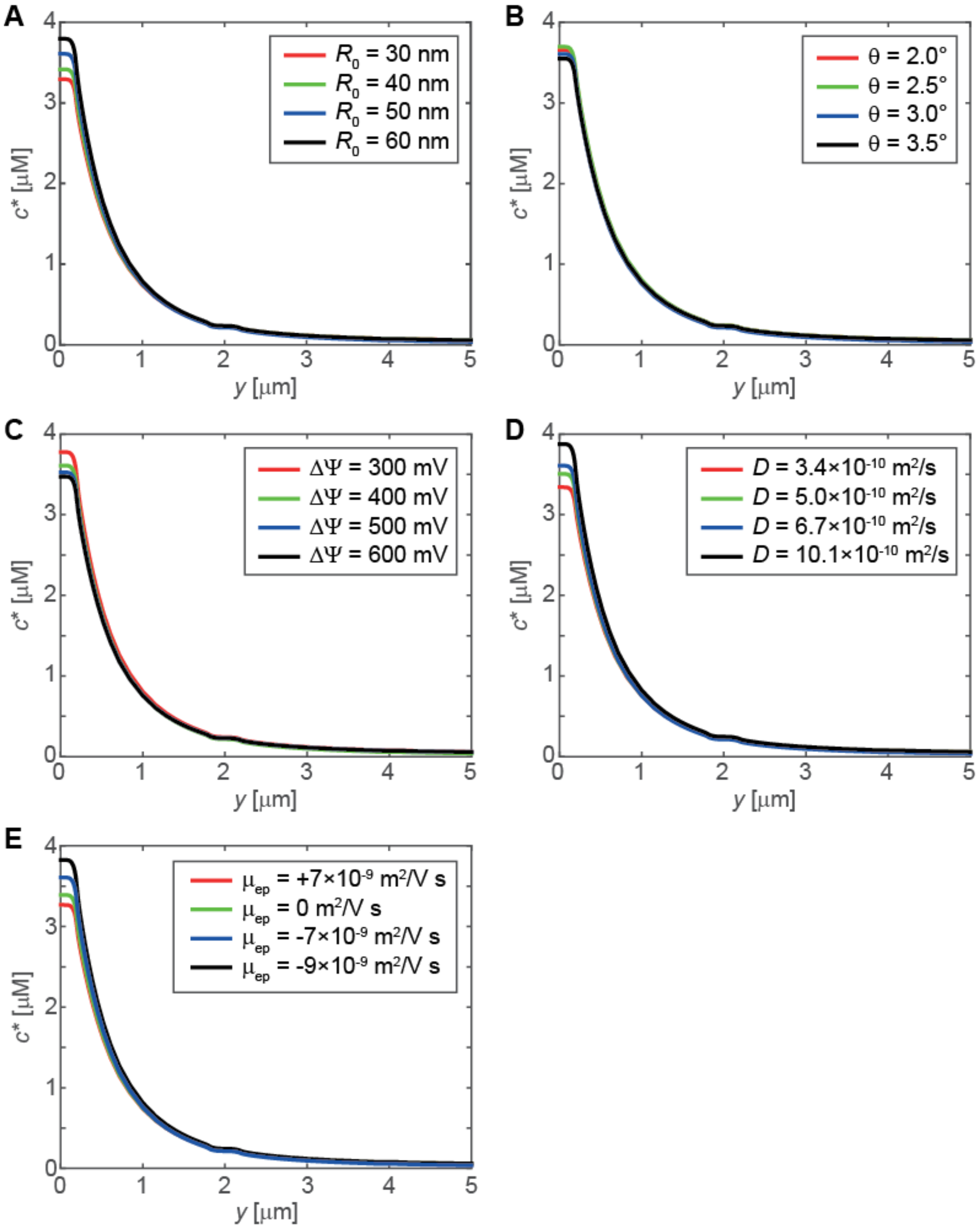

Supporting Figure 3. Various normalized concentration profiles where one parameter at a time has been varied and the other parameters have the value in Table 1. The data is normalized to the parameter value in Table 1 based on the expression in Eq. 3, which for the different situations was done by setting: **(A)** $c^* = c\times((50\text{ nm})/R_0)$, **(B)** $c^* = c\times(\tan(3°)/\tan(\theta))$, **(C)** $c^* = c\times((400\text{ mV})/\Delta\Psi)$, **(D)** $c^* = c\times(D/(6.7\times10^{-10}\text{ m}^2/\text{s}))$ and **(E)** $c^* = c\times((7\times10^{-9}\text{ m}^2/\text{V s})/(\mu_{ep}+14\times10^{-9}\text{ m}^2/\text{V s}))$.

Additional simulations were also performed where the height, h, between the pipette and the T-tubule opening was varied under otherwise the same conditions as in Table 1. The obtained concentration profiles, c, normalized with the corresponding concentration profiles from Eqs. 1 and 2, c_{flat}, are given in Supporting Figure 4. It can be observed that the concentration at the T-tubule opening increases faster relative to delivery to a flat surface as the pipette approaches the T-tubule opening. When the pipette is far above the Z-groove the concentration profile approaches the values obtained for a flat surface.

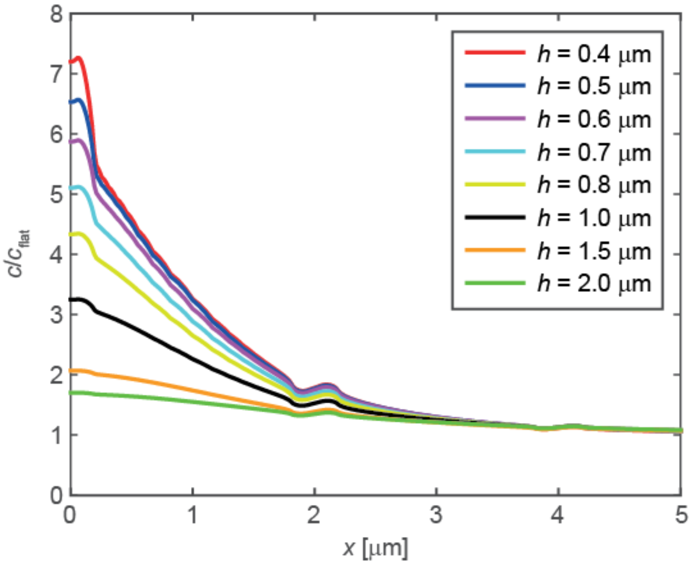

Supporting Figure 4. Concentration profiles at different distances, h, between the tip of the pipette and the T-tubule opening normalized with the theoretical expression in Eqs. 1 and 2 for the concentration profile when delivering to a flat surface. All other parameter values were the same as in Table 1.

Supporting References:

[1] Sato M, O'Gara P, Harding SE, Fuller SJ. (2005) Enhancement of adenoviral gene transfer to adult rat cardiomyocytes in vivo by immobilization and ultrasound treatment of the heart. *Gene Ther*. 12(11):936-41.

[2] Nikolaev VO, Moshkov A, Lyon AR, Miragoli M, Novak P, Paur H, Lohse MJ, Korchev YE, Harding SE, Gorelik J. (2010) Beta2-adrenergic receptor redistribution in heart failure changes cAMP compartmentation. *Science*, 327(5973):1653-7.

[3] Novak P, Li C, Shevchuk AI, Stepanyan R, Caldwell M, Hughes S, Smart TG, Gorelik J, Ostanin VP, Lab MJ, Moss GW, Frolenkov GI, Klenerman D, Korchev YE. (2009) Nanoscale live-cell imaging using hopping probe ion conductance microscopy. *Nat. Methods* 6(4):279-81.

[4] Edelstein A.D, Tsuchida MA, Amodaj N, Pinkard H, Vale RD, Stuurman N. (2014) Advanced methods of microscope control using μManager software. *J. Biol. Methods* 1(2):e10.

[5] Babakinejad B., Jönsson P, López Córdoba A, Actis P, Novak P, Takahashi Y, Shevchuk A, Anand U, Anand P, Drews A, Ferrer-Montiel A, Klenerman D, Korchev YE. (2013) Local delivery of molecules from a nanopipette for quantitative receptor mapping on live cells. *Anal. Chem.* 85(19):9333-42.
